# Supplementary material for: Imitation recognition and its prosocial effects in 6-month old infants
Source: PLoS One. 2020 May 20;15(5):e0232717. doi: 10.1371/journal.pone.0232717 (PMC7239450; doi:10.1371/journal.pone.0232717)
Supplement: S1 Data — (DOCX) [file pone.0232717.s001.docx]

**1.PRELIMINARY ANALYSES FOR ASSESSING THE EFFECTS OF ORDER ON ATTENTION, SMILING, APPROACH AND TESTING BEHAVIOURS DURING THE EXPERIMENTAL CONDITIONS**

To determine the effects of order on the dependent variables, one-way repeated-measures ANOVAs were conducted with order (i.e. experimental slot) as a within-subjects factor. Whenever parametric test assumptions were violated, and data transformation did not improve normality, non-parametric ANOVAs were instead conducted. Whenever necessary, relevant follow-up tests are conducted. To control for false discovery rates (Type I error), p-values for multiple comparisons were corrected using the Benjamini-Hochberg procedure. P-value corrections, however, were not carried out when all p-values in each set of multiple comparisons were non-significant. Given the small sample, the Shapiro-Wilk test was employed to determine if data differed significantly from a normal distribution. For the same reason, exact significance (two-tailed) is always reported for the non-parametric tests.

Sex was added as a between-subjects factor in order to assess potential interaction effects between condition and sex on the dependent variables.

**1.1. THE EFFECTS OF ORDER ON ATTENTION DURING THE EXPERIMENTAL CONDITIONS**

**1.1.1. Effects of order on duration of attention during the experimental conditions**

Shapiro-Wilk tests indicated that the normality assumption was met for all four slots (W_Slot1_ = 0.911, p = 0.12; W_Slot2_ = 0.918, p = 0.156; W_Slot3_ = 0.931, p = 0.250; W_Slot4_ = 0.928, p = 0.229). Moreover, Mauchly's Test of Sphericity indicated that the sphericity assumption was also met: *χ*^2^(5)= 3.960; p = 0.556. A repeated-measures ANOVA was thus conducted to assess the effects of order on the overall duration of attention, which yielded non-significant results: F(3,45) = 1.763, p = 0.168. Thus, no follow-up focused comparisons were done. In addition, we also conducted a mixed- model ANOVA to assess the effects of order and sex on the overall duration of attention. Mauchly's Test of Sphericity showed that the sphericity assumption was also met: *χ^2^*(5) = 6.331; p = 0.277. The results of this test were non-significant. There was a non-significant main effect of order (F(3,42) = 0.764, p = 0.521), a non-significant main effect of sex (F(1,14) = 0.148, p = 0.706), and a non-significant interaction effect between order and sex: F(3,42) = 1.417, p = 0.251.

**1.1.2. Effects of order on the frequency of attention bouts during the experimental conditions**

Shapiro-Wilk tests indicated that the normality assumption was violated for one group (i.e. slot 4): W_Slot1_ = 0.946, p = 0.434; W_Slot2_ = 0.926, p = 0.211; W_Slot3_ = 0.971, p = 0.862; W_Slot4_ = 0.820, p = 0.005. To address this, the data were subjected to logarithmic transformation, which improved normality for all slots: W_Slot1_ = 0.944, p = 0.404; W_Slot2_ = 0.949, p = 0.467; W_Slot3_ = 0.929, p = 0.233; W_Slot4_ = 0.893, p = 0.061. Subsequent analyses were thus conducted on log transformed data. Mauchly's Test of Sphericity showed that the sphericity assumption was met: *χ*^2^(5)= 8.478; p = 0.133. The results of the repeated-measures ANOVA, conducted to assess the effects of order on the frequency of attention bouts, were non-significant (F(3,45) = 1.084, p = 0.366), and thus no follow-up tests conducted. In addition, we conducted a mixed-model ANOVA, with order as a within-subject factor and sex as a between-subjects variable, which yielded no significant main effects of order (F(3,42) = 0.346, p = 0.792) or sex (sex: F(1,14) = 0.054, p = 0.819). As reported in the Results section of the manuscript, this analysis revealed a significant interaction between order and sex: F(3,42) = 3.097, p = 0.037, η_p_^2^ = 0.181. Follow-up comparisons using the independent t-test were conducted for each of the four data sets, but all results were non-significant. As such, for slot 1: t(14) = -1.260, p = 0.228; for slot 2: t(4.451) = 0.836, p = 0.445; for slot 3: t(14) = 1.026, p = 0.322; for slot 4: t(4.715) = -1.252, p = 0.269 (Note that these are uncorrected p-values, and the degree of freedom are adjusted for slot 2 and slot 4, as the homogeneity of variance assumption was violated for these.

**1.1.3.** **Effects of order on average duration of attention bouts during the experimental conditions**

Shapiro-Wilk tests indicated that the normality assumption was violated for one slot (i.e. slot 3): W_Slot1_ = 0.934, p = 0.286; W_Slot2_ = 0.912, p = 0.124; W_Slot3_ = 0.834, p = 0.008; W_Slot4_ = 0.901, p = 0.085. A logarithmic transformation improved normality (W_Slot1_ = 0.978, p = 0.943; W_Slot2_ = 0.956, p = 0.582; W_Slot3_ = 0.912, p = 0.124; W_Slot4_ = 0.921, p = 0.173), and thus, in order to assess the effect of order on the average duration of attention bouts, a repeated-measure ANOVA was conducted on log transformed data, with order as the within-subjects factor. Mauchly's Test of Sphericity indicated that the sphericity assumption was met: *χ*^2^(5)= 10.477; p = 0.063. The results of the ANOVA were non-significant (F(3,45) = 1.475, p = 0.234), and thus no follow-up comparisons were conducted. We also conducted a mixed-model ANOVA, with order as the within-subjects factor and sex as a between-subjects factor. Mauchly's Test of Sphericity showed that the sphericity assumption was met: *χ^2^*(5)= 10.436; p = 0.064. The results of the ANOVA were non-significant. There was a non-significant main effect of order (F(3,42) = 0.382, p = 0.766), a non-significant main effect of sex (F(1,14) = 0.001, p = 0.973), and a non-significant interaction effect between order and sex: F(3,42) = 2.457, p = 0.076.

**1.2. THE EFFECTS OF ORDER ON SMILING DURING THE EXPERIMENTAL CONDITIONS**

**1.2.1. Effects of order on the overall duration of smiling during the experimental conditions**

Shapiro-Wilk tests indicated that the normality assumption was violated for one slot (i.e. slot 2): W_Slot1_ = 0.965, p = 0.760; W_Slot2_ = 0.848, p = 0.013; W_Slot3_ = 0.925, p = 0.207; W_Slot4_ = 0.877, p = 0.035. While logarithmic transformation did not have the effect of improving data normality, normality was improved by square root transformation: W_Slot1_ = 0.954, p = 0.564; W_Slot2_ = 0.929, p = 0.235; W_Slot3_ = 0.968, p = 0.810; W_Slot4_ = 0.952, p = 0.521. To assess the effects of order on the total duration of smiling, a repeated-measures ANOVA was thus conducted on square root transformed data, with order as a within-subjects factor. Mauchly's Test of Sphericity showed that the sphericity assumption was met: *χ*^2^(5)= 10.634; p = 0.060. The results of the ANOVA were non-significant: F(3,45) = 1.804, p = 0.160**.** In addition, a mixed-model ANOVA with order as a within-subjects factor and sex as a between-subjects factor was thus conducted on square root transformed data. Mauchly's Test of Sphericity indicated that the sphericity assumption was met: *χ^2^*(5) = 10.362; p = 0.066**.** The results of the mixed-model ANOVA were too non-significant. There was a non-significant main effect of order (F(3,42) = 0.873, p = 0.463), a non-significant main effect of sex (F(1,14) = 2.232, p = 0.157), and a non-significant interaction effect between order and sex: F(3,42) = 0.896, p = 0.451.

**1.2.2. Effects of order on the frequency of smiling bouts during the experimental conditions**

Shapiro-Wilk tests indicated that the normality assumption was met for all four slots: W_Slot1_ = 0.955, p = 0.571; W_Slot2_ = 0.934, p = 0.284; W_Slot3_ = 0.962, p = 0.704; W_Slot4_ = 0.955, p = 0.578. To assess the effect of order on the frequency of smiling bouts, a repeated-measures ANOVA was conducted with order as the within-subject factor. Mauchly's Test of Sphericity indicated that the sphericity assumption was also met: *χ*^2^(5)= 7.186; p = 0.208. The results of the ANOVA were non-significant (F(3,45) = 2.091, p = 0.115), and thus no follow-up comparisons were conducted. We also conducted a mixed-model ANOVA with order as the within-subjects factor and sex as the between-subjects factor. Mauchly's Test of Sphericity indicated that the sphericity assumption was also met: *χ^2^*(5) = 7.127; p = 0.213. The ANOVA yielded non-significant results: there was a non-significant main effect of order (F(3,42) = 1.376, p = 0.263), a non-significant main effect of sex (F(1,14) = 0.024, p = 0.878) and a non-significant interaction effect between sex and order: F(3,42) =0.395, p = 0.757.

**1.2.3. Effects of order on the average duration of smiling bouts during the experimental conditions**

Shapiro-Wilk tests indicated that the normality assumption was violated for one slot (i.e. slot 4): W_Slot1_ = 0.962, p = 0.705; W_Slot2_ = 0.937, p = 0.311; W_Slot3_ = 0.906, p = 0.102; W_Slot4_ = 0.822, p = 0.005. A logarithmic transformation improved normality: W_Slot1_ = 0.897, p = 0.073; W_Slot2_ = 0.972, p = 0.870; W_Slot3_ = 0.972, p = 0.865; W_Slot4_ = 0.955, p = 0.579. To assess the effects of order on the average duration of smiling bouts, a repeated-measures ANOVA was conducted on log transformed data, with order as the within-subjects factor. Mauchly's Test of Sphericity indicated that the sphericity assumption was met: *χ*^2^(5)= 7.720; p = 0.173. The results of the ANOVA were non-significant (F(3,45) = 0.699, p = 0.557), and thus no follow-up tests were conducted. We also ran a mixed-model ANOVA with order as the within-subjects factor and sex as a between-subjects factor. Mauchly's Test of Sphericity indicated no violation of the sphericity assumption: *χ^2^*(5) = 7.152; p = 0.211. The results of the ANOVA were non-significant: there was a non-significant main effect of order (F(3,42) = 0.556, p = 0.647), a non-significant main effect of sex (F(1,14) = 3.896, p = 0.068), and a non-significant interaction effect between order and sex (F(3,42) = 0.895, p = 0.452).

**1.3. THE EFFECTS OF ORDER ON APPROACH DURING THE EXPERIMENTAL CONDITIONS**

**1.3.1. Effects of order on overall duration of approach during the experimental conditions**

Shapiro-Wilk tests indicated that the normality assumption was violated for all four slots: W_Slot1_ = 0.817, p = 0.005; W_Slot2_ = 0.691, p < 0.000; W_Slot3_ = 0.724, p < 0.000; W_Slot4_ = 0.750, p = 0.001. Since normality was not sufficiently improved by data transformation, a Friedman’s ANOVA was conducted to assess the effects of order on the total duration of approach behaviours. The result of this test was non-significant (*χ^2^*(3) = 2.689, p = 0.453), and thus no follow-up tests were conducted.

**1.3.2. Effects of order on the frequency of approach bouts during the experimental conditions**

Shapiro-Wilk tests indicated that the normality assumption was violated for three slots: W_Slot1_ = 0.890, p = 0.055; W_Slot2_ = 0.818, p = 0.005; W_Slot3_ = 0.763, p = 0.001; W_Slot4_ = 0.853, p = 0.015. Since normality was not improved by data transformation, a Friedman’s ANOVA was conducted to assess the effects of order on the frequency of approach bouts. Since the result of this test was non-significant (*χ^2^*(3) = 4.042, p = 0.261), no follow-up comparisons were conducted.

**1.3.3. Effects of order on the average duration of approach bouts during the experimental conditions**

Shapiro-Wilk tests indicated that the normality assumption was violated for three slots: W_Slot1_ = 0.911, p = 0.123; W_Slot2_ = 0.861, p = 0.020; W_Slot3_ = 0.675, p < 0.000; W_Slot4_ = 0.771, p = 0.001. Since normality was not improved by data transformation, a Friedman’s ANOVA was conducted to assess the effects of order on the average duration of approach bouts. This test yielded non-significant results (*χ^2^*(3) = 2.067, p = 0.572), and thus no follow-up tests were done.

**1.4. THE EFFECTS OF ORDER ON TESTING**

**1.4.1. Effects of order on the overall duration of testing behaviours**

Shapiro-Wilk tests indicated that the normality assumption was violated for all slots: W_Slot1_ = 0.869, p = 0.026; W_Slot2_ = 0.785, p = 0.002; W_Slot3_ = 0.839, p = 0.009; W_Slot4_ = 0.854, p = 0.016. Normality was not improved by data transformation, and thus a Friedman’s ANOVA was conducted to assess the effects of order on the overall duration of testing. The results of this test were non-significant (*χ^2^*(3) = 0.869, p = 0.844), and thus no follow-up tests were conducted.

**1.4.2. Effects of order on the frequency of testing bouts**

Shapiro-Wilk tests indicated that the normality assumption was violated for all slots: W_Slot1_ = 0.875, p = 0.033; W_Slot2_ = 0.805, p = 0.003; W_Slot3_ = 0.800, p = 0.003; W_Slot4_ = 0.742, p = 0.001. Normality was not improved by data transformation, and thus a Friedman’s ANOVA was conducted to assess the effects of order on the frequency of testing bouts. The results of the test were non-significant (*χ^2^*(3) = 0.993, p = 0.813) and thus no follow-up comparisons were done.

**1.4.3. Effects of order on latency to the first testing bout**

Shapiro-Wilk tests indicated that the normality assumption was violated for all slots: W_Slot1_ = 0.720, p < 0.001; W_Slot2_ = 0.827, p = 0.006; W_Slot3_ = 0.752, p = 0.001; W_Slot4_ = 0.766, p = 0.001. Since normality was not sufficiently improved by data transformation, a Friedman’s ANOVA was conducted to assess the effects of order on latency to the first testing bout. The results of this test were non-significant (*χ^2^*(3) = 0.207, p = 0.978), and thus no follow-up tests were conducted.

**2. THE EFFECTS OF SEX ON THE DEPENDENT VARIABLES DURING THE EXPERIMENTAL CONDITIONS - NON-SIGNIFICANT RESULTS**

To assess the effects of sex on the 12 dependent variables, independent t-test were conducted, supplanted by the Mann-Whitney U test whenever data failed to meet parametric test assumptions. To further capture potential interaction effects between sex and order, or between sex and condition, additional tests (independent t-tests or Mann-Whitney U tests) were conducted separately for each experimental slot and each experimental condition, with sex as the grouping variable.

**2.1. THE EFFECTS OF SEX ON ATTENTION DURING THE EXPERIMENTAL CONDITIONS**

**2.1.1. The effects of sex on the total duration of attention during the experimental conditions**

Shapiro-Wilks tests indicated that the normality assumption was violated for the male group: W_Males_ = 0.924, p = 0.006; W_Females_ = 0.945, p = 0.297. Since normality was not sufficiently improved by data transformation, the Mann-Whitney U test was conducted to assess the effects of sex on the total duration of attention. The results of this test were non-significant: U = 416.000, p = 0.736. The effect of sex on the total duration of attention was further assessed separately, for each order slot and for each condition. These tests too yielded non-significant results: U_Slot 1_ = 18.000, p = 0.320; U_Slot 2_ = 15.000, p = 0.180; U_Slot 3_ = 19.000, p = 0.377; U_Slot 4_ = 27.000, p = 1, U_MI_ = 27.000, p = 1; U_CI_ = 24.000, p = 0.743; U_BI_ = 26.000, p = 0.913; U_CR_ = 22.000, p = 0.583.

**2.1.2. The effects of sex on the frequency of attention bouts during the experimental conditions**

Shapiro-Wilks tests indicated that the normality assumption was violated for the female group: W_Males_ = 0.972, p = 0.363; W_Females_ = 0.877, p = 0.016. Since data transformation did not improve normality, we conducted a Mann-Whitney U test to assess the effect of sex on the frequency of attention bouts, which yielded non-significant results: U=436.500, p = 0.963. Separate Mann-Whitney U tests were conducted to further assess the effect of sex in each experimental slot and each condition. All these tests yielded non-significant results: U_Slot 1_ = 17.500, p = 0.274; U_Slot 2_ = 18.000, p = 0.299; U_Slot 3_ = 16.500, p = 0.233; U_Slot 4_ = 17.000, p = 0.250; U_MI_ = 24.000, p = 0.723; U_CI_ = 26.500, p = 0.937; U_BI_ = 27.000, p = 0.974; U_CR_ = 23.000, p = 0.633.

**2.1.3. The effects of sex on the average duration of attention bouts during the experimental intervals**

Shapiro-Wilks tests indicated that the normality assumption was violated for both groups: W_Males_ = 0.916, p = 0.004; W_Females_ = 0.895, p = 0.033. Logarithmic transformation improved normality: W_Males_ = 0.977, p = 0.504; W_Females_ = 0.916, p = 0.085. Thus, to assess the effect of sex on the average duration of attention bouts an independet t-test was conducted. The results of this test were non-significant: *t*(30.5) = -0.03, p = 0.976. Separate tests were further conducted to assess the effects of sex on the average duration of attention bouts in each experimental slot. Since the data in several groups violated the normality assumption, a logarithmic transformation was conducted, which improved data normality for all eight groups. Independent t-test were subsequently conducted, and all yielded non-significant results: Slot 1: t(14) = 1.768, p = 0.099; slot 2: t(14) = -1.530, p = 0.148; slot 3: t(5.3) = -0.871, p = 0.422, and slot 4: t(14) = 0.957, p = 0.355. Note that the degrees of freedom for slot 3 are adjusted, since slot 3 data did not meet homogeneity assumptions. Additional tests were also conducted to separately assess the effect of sex on the average duration of attention bout for each condition. Since the normality assumption was violated for several groups, and data transformation failed to sufficiently improve normality, four Mann-Whithney tests were carried out for this purpose. All these tests yielded non-significant results: U_MI_ = 26.000, p = 0.913; U_CI_ = 26.000, p = 0.913; U_BI_ = 22.000, p = 0.583; U_CR_ = 23.000, p= 0.661.

**2.2. THE EFFECTS OF SEX ON SMILING DURING THE EXPERIMENTAL CONDITIONS**

**2.2.1. The effects of sex on the total duration of smiling during the experimental conditions**

Shapiro-Wilks tests indicated that the normality assumption was violated for both groups: W_Males_ = 0.923, p = 0.006; W_Females_ = 0.885, p = 0.022. While logarithmic transformation did not sufficiently improve normality, square root transformation did: W_Males_ = 0.990, p = 0.965; W_Females_ = 0.962, p = 0.590. To assess the effect of sex on the total duration of smiling, an independet t-test was thus conducted, which yielded non-significant results: t(62) = 1.914, p = 0.060. Additional independent t-tests were conducted to assess the effects of sex on the total duration of smiling separately for each experimental slot and each experimental condition. All these tests were carried out on log transformed data, as the normality assumption - although initially violated - improved sufficiently following logarithmic transformation. As such: slot 1: t(4.7) = 1.428, p = 0.217; slot 2: t(14) = -0.397, p = 0.698; slot 3: t(14) = 1.924, p = 0.075; slot 4: t(14) = 0.691, p = 0.501; MI condition: t(14) = 1.693, p = 0.112; CI condition: t(4.9) = 1.136, p = 0.309; BI condition: t(14) = 0.325, p = 0.750; CR condition: t(14) = 0.928, p = 0.369 (note that the degrees of freedom are adjusted for slot 1, as well as for the CI condition as the data from those groups violated the homogeneity assumption).

**2.2.2. The effects of sex on the frequency of smiling bouts during the experimental conditions**

Shapiro-Wilks tests indicated that the normality assumption was met for both groups: W_Males_ = 0.961, p = 0.143; W_Females_ = 0.950, p = 0.365. To assess the effect of sex on the frequency of smiling bouts, an independent t-test was conducted, which yielded non-significant results: t(62) = - 0.208, p = 0.836. In addition, independent t-tests were conducted to assess the effects of sex on the total duration of smiling separately for each experimental slot and for each condition. All these tests yielded non-significant results. As such, slot 1: t(14) = 0.435, p = 0.670; slot 2: t(14) = - 0.519, p = 0.612; slot 3: t(14) = 0.196, p = 0.847; slot 4: t(14) = -0.669, p = 0.514, as well as MI condition: t(14) = 0.977, p = 0.345; CI condition: t(4.6) = -0.165, p = 0.876; BI condition: t(14) = -0.947, p = 0.360; CR condition: t(14) = 0.167, p = 0.869 (note that the degrees of freedom for the CI condition are adjusted as data from this group violated the homogeneity assumption).

**2.2.3. The effects of sex on the average duration of smiling bouts during the experimental conditions**

As reported in the manuscript, we found a significant effect of sex on the average duration of smiling bouts: t(62) = 2.737, p = 0.008. Since Shapiro-Wilks tests indicated that the normality assumption was violated for the male group (W_Males_ = 0.887, p < 0.001; W_Females_ = 0.970, p = 0.749), and logarithmic transformation improved normality, (W_Males_ = 0.960, p = 0.131; W_Females_ = 0.950, p = 0.364) this analysis was conducted on log transformed data. Independent t-tests were further conducted on log transformed data to assess the effects of sex on the average duration of smiling bouts separately, for each order slot. These analyses yielded non-significant results: t(14) = 2.624, p = 0.080 (uncorrected p = 0.029), slot 2: t(14) = 0.248, p = 0.808 (uncorrected p = 0.808); slot 3: t(14) = 1.537, p = 0.216 (uncorrected p = 0.147); slot 4: t(14) = 1.475, p = 0.216 (uncorrected p = 0.162). In addition, to assess the effect of sex on the average duration of smiling bouts separately for each condition, four Mann-Whitney U tests were conducted (as data did not meet the normality assumption, and normality was not sufficiently improved by transformation). The results of all these tests were non-significant: U_MI_ = 17.000, p = 0.267; U_CI_ = 12.000, p = 0.090; U_BI_ = 17.500, p = 0.278; U_CR_ = 12.000, p= 0.090.

**2.3. THE EFFECTS OF SEX ON APPROACH BEHAVIOURS DURING THE EXPERIMENTAL CONDITIONS**

**2.3.1. The effects of sex on the total duration of approach behaviours during the experimental conditions**

Shapiro-Wilks tests indicated that the normality assumption was violated for both groups: W_Males_ = 0.742, p < 0.001; W_Females_ = 0.785, p = 0.001. Since normality was not improved by data transformation, a Mann-Whitney U test was conducted to assess the effect of sex on approach behaviours, which yielded non-significant results: U = 431.000, p = 0.902. To assess the effect of sex on approach duration separately for each experimental slot and for each experimental condition, eight Mann-Whitney tests were conducted. The results of all these tests were non-significant: U_Slot 1_ = 27.000, p = 1; U_Slot 2_ = 22.500, p = 0.601; U_Slot 3_ = 26.000, p = 0.909; U_Slot 4_ = 20.000, p = 0.432; U_MI_ = 22.000, p = 0.582; U_CI_ = 24.000, p = 0.716; U_BI_ = 24.000, p = 0.738; U_CR_ = 22.000, p= 0.567.

**2.3.2. The effects of sex on the frequency of approach bouts during the experimental conditions**

Shapiro-Wilks tests indicated that the normality assumption was violated for both groups: W_Males_ = 0.857, p < 0.001; W_Females_ = 0.817, p = 0.002. Data transformation did not improve normality, and thus a Mann-Whitney U test was conducted to assess the effects of sex on the frequency of approach bouts. This test yielded non-significant results: U = 394.000, p = 0.502. To assess the effect of sex on the frequency of approach in each experimental slot and each experimental condition, eight Mann-Whitney tests were conducted. The results of all these tests were non-significant: U_Slot 1_ = 21.000, p = 0.490; U_Slot 2_ = 21.500, p = 0.546; U_Slot 3_ = 23.500, p = 0.694; U_Slot 4_ = 18.000, p = 0.298; U_MI_ = 24.500, p = 0.761; U_CI_ = 22.000, p = 0.559; U_BI_ = 22.500, p = 0.601; U_CR_ = 18.500, p= 0.315.

**2.3.3. The effects of sex on the average duration of approach bouts during the experimental conditions**

Shapiro-Wilks tests indicated that the normality assumption was severely violated for both groups: W_Males_ = 0.748, p < 0.001; W_Females_ = 0.785, p = 0.001. Since normality was not improved by data transformation, a Mann-Whitney U test was conducted to assess the effects of sex on the average duration of approach bouts. The test yielded non-significant results: U = 417.000, p = 0.743. To assess the effect of sex on the average duration of approach boutsseparately, for each experimental slot and each experimental condition, eight Mann-Whitney tests were conducted. The results of all these tests were non-significant: U_Slot 1_ = 23.000, p = 0.660; U_Slot 2_ = 21.500, p = 0.527; U_Slot 3_ = 25.000, p = 0.817; U_Slot 4_ = 25.000, p = 0.825; U_MI_ = 16.000, p = 0.220; U_CI_ = 26.000, p = 0.886; U_BI_ = 27.000, p = 1; U_CR_ = 24.000, p = 0.734.

**2.4. THE EFFECTS OF SEX ON TESTING BEHAVIOURS**

**2.4.1. The effects of sex on the total duration of testing behaviours**

Shapiro-Wilks tests indicated that the normality assumption was severely violated for both groups: W_Males_ = 0.863, p < 0.001; W_Females_ = 0.809, p = 0.001. Since normality was not improved by data transformation, a Mann-Whitney U test was conducted to assess the effects of sex on the total duration of testing behaviours. The results of the test were non-significant: U = 385.500, p = 0.428. To assess the effects of sex on testing duration separately, for each experimental slot and each experimental condition, eight Mann-Whitney tests were conducted, which all yielded non-significant results: U_Slot 1_ = 22.000, p = 0.577; U_Slot 2_ = 22.500, p = 0.601; U_Slot 3_ = 18.500, p = 0.329; U_Slot 4_ = 20.000, p = 0.432; U_MI_ = 26.000, p = 0.913; U_CI_ = 27.000, p = 1; U_BI_ = 19.500, p = 0.385; U_CR_ = 18.000, p = 0.245.

**2.4.2. The effects of sex on the frequency of testing bouts**

Shapiro-Wilks tests indicated that the normality assumption was severely violated for both groups: W_Males_ = 0.868, p < 0.001; W_Females_ = 0.777, p < 0.001. Normality was not improved by data transformation and, thus, to assess for the effects of sex on the frequency of testing bouts a Mann-Whitney U test was conducted, which yielded non-significant results: U = 413.000, p = 0.693. The effects of sex on testing frequency was also assessed separately for each experimental slot and each experimental condition, but the results of the eight Mann-Whitney U tests conducted for this purpose, were non-significant: U_Slot 1_ = 22.000, p = 0.557; U_Slot 2_ = 24.500, p = 0.759; U_Slot 3_ = 19.500, p = 0.389; U_Slot 4_ = 23.000, p = 0.676; U_MI_ = 22.500, p = 0.591; U_CI_ = 25.500, p = 0.847; U_BI_ = 24.500, p = 0.777; U_CR_ = 20.000, p = 0.540.

**2.4.3. The effects of sex on the latency to the first testing bout**

Shapiro-Wilks tests indicated that the normality assumption was severely violated for both groups: W_Males_ = 0.774, p < 0.001; W_Females_ = 0.729, p < 0.001. Normality was not improved by data transformation and, thus, to assess for the effects of sex on latency to the first testing bout, a Mann-Whitney U test was conducted, which yielded non-significant results: U = 412.000, p = 0.685. To assess the effects of sex on latency to the first testing bout separately, for each experimental slot and each experimental condition, eight Mann-Whitney tests were conducted, which all yielded non-significant results: U_Slot 1_ = 26.000, p = 0.884; U_Slot 2_ = 20.000, p = 0.418; U_Slot 3_ = 17.500, p = 0.260; U_Slot 4_ = 20.500, p = 0.450; U_MI_ = 12.500, p = 0.096; U_CI_ = 22.000, p = 0.563; U_BI_ = 19.000, p = 0.357; U_CR_ = 21.500, p = 0.530.

**3. THE EFFECTS OF CONDITION ON THE DEPENDENT VARIABLES DURING THE EXPERIMENTAL CONDITIONS - NON-SIGNIFICANT RESULTS**

To determine the effects of condition on the dependent variables, one-way repeated-measures ANOVAs were conducted with condition as a within-subjects factor. Whenever parametric test assumptions were violated, and data transformation did not improve normality, non-parametric ANOVAs were instead conducted. Whenever the ANOVAs yielded significant results, relevant follow-up tests were carried out. In addition, to assess interaction effects between condition and sex mixed-model ANOVAs were also conducted with condition as the within-subject factor and sex as a between-subjects factor. To control for false discovery rates (Type I error), p-values for all multiple comparisons were corrected using the Benjamini-Hochberg procedure. P-value corrections, however, were not carried out when all p-values in each set of multiple comparisons were non-significant. Given the small sample, the Shapiro-Wilk test was employed to determine if data differed significantly from a normal distribution. For the same reason, exact significance (two-tailed) is always reported for the non-parametric tests.

**3.1. THE EFFECTS OF CONDITION ON ATTENTION DURING THE EXPERIMENTAL CONDITIONS**

**3.1.1. Effects of condition of overall duration of attention during the experimental conditions**

Shapiro-Wilk tests indicated that the normality assumption was violated for the MI condition**:** W_MI_ = 0.854, p = 0.016; W_CI_ = 0.957, p = 0.599; W_BI_ = 0.966, p = 0.765; W_CR_ = 0.894, p = 0.064. Since normality was not improved by data transformation, a Friedman’s ANOVA was conducted to assess the effects of condition on attention duration. As reported in the Results section of the manuscript, the results of this test were significant: *χ^2^*(3) = 25.575, p < 0.001. Follow-up paired comparisons based on the Wilcoxon Signed-Rank test yielded, besides the significant results reported in the manuscript, the following non-significant result for MI vs. CI: Z = -0.776, p = 0.464 (uncorrected p-value = 0.464).

**3.1.2. Effects of condition on the frequency of attention bouts during the experimental conditions**

Shapiro-Wilk tests indicated that the normality assumption was violated for the MI and CR conditions: W_MI_ = 0.884, p = 0.045; W_CI_ = 0.922, p = 0.181; W_BI_ = 0.952, p = 0.519; W_CR_ = 0.881, p = 0.040. Logarithmic transformation improved data normality: W_MI_ = 0.954, p = 0.548; W_CI_ = 0.922, p = 0.181; W_BI_ = 0.944, p = 0.401; W_CR_ = 0.929, p = 0.235. To assess the effects of condition on the frequency of attention bouts, a one-way repeated measures ANOVA was thus conducted on the log transformed data, with condition as the repeated factor. As reported in the manuscript, sphericity assumption for this test were met (Mauchly's Test of Sphericity: *χ^2^*(5) = 1.191; p = 0.946) and the results of this test were significant: F(3,45) = 6.059, p = 0.001. A supplementary mix-model ANOVA was also conducted (on log transformed data) with condition as a within-subjects factor and sex as a between-subjects factor. Mauchly's Test of Sphericity indicated that the sphericity assumption was met (*χ^2^*(5) = 1.540; p = 0.909), and the results of this test also revealed a significant main effect of condition (F(3,42) = 5.807, p = 0.002, η_p_^2^ = 0.293), but no other significant effects for sex (F(1,14) = 0.054, p = 0.819) or for the condition and sex interaction: F(3,42) = 0.404, p = 0.751. Follow-up comparisons using the paired t-test were conducted, yielding besides the significant results reported in the manuscript, the following non-significant results: MI vs. CI: *t(*15) = -0.938, p = 0.436 (uncorrected p = 0.363); CI vs. BI: *t*(15) = -2.007, p = 0.095 (uncorrected p = 0.063), and BI vs. CR: *t*(15) = - 0.607, p = 0.553 (uncorrected p = 0.553).

**3.1.3. The effects of condition on the average duration of attention bouts during the experimental conditions**

Shapiro-Wilk tests indicated that the normality assumption was violated for the CI and CR conditions: W_MI_ = 0.943, p = 0.383; W_CI_ = 0.858, p = 0.018; W_BI_ = 0.905, p = 0.095; W_CR_ = 0.785, p = 0.002. As data transformation did not improve normality, a Friedman’s ANOVA was conducted to assess the effect of condition on the average duration of attention bouts. As reported in the manuscript, the results of this test were significant: *χ^2^*(3) = 25.875, p < 0.001. Follow-up paired comparisons were conducted using the Wilcoxon signed-rank test, which yielded, besides the significant results reported in the manuscript, the following non-significant results: MI vs. CI: Z = -1.086, p = 0.298 (uncorrected p = 0.298) and BI vs. CR: Z = -1.655, p = 0.126 (uncorrected p = 0.105).

**3.2. THE EFFECTS OF CONDITION ON SMILING DURING THE EXPERIMENTAL CONDITIONS**

**3.2.1. The effects of condition on the overall duration of smiling during the experimental conditions**

Shapiro-Wilk tests indicated that the normality assumption was violated for the BI and CR conditions: W_MI_ = 0.908, p = 0.107; W_CI_ = 0.917, p = 0.149; W_BI_ = 0.877, p = 0.035; W_CR_ = 0.826, p = 0.006. Logarithmic transformation improved data normality: W_MI_ = 0.940, p = 0.347; W_CI_ = 0.968, p = 0.804; W_BI_ = 0.912, p = 0.125; W_CR_ = 0.980, p = 0.961. To assess the effects of condition on the duration of smiling, a one-way repeated-measures ANOVA was thus conducted on log transformed data with condition as the within-subjects effect. As reported in the manuscript, the results of this test were significant: F (2.1; 31.5) = 5.777, p = 0.007, η_p_^2^ = 0.278. (Note that degrees of freedom are adjusted, since the sphericity assumption was not met: *χ*^2^(5) = 13.448; p = 0.020). A supplementary mixed-model ANOVA was further conducted, with condition as a within-subjects factor and sex as a between-subjects factor. The sphericity assumption was violated for this test too (Mauchly's Test of Sphericity: *χ^2^*(5)= 12.382; p = 0.030), and thus degrees of freedom were corrected using the Greenhouse-Geisser estimate of sphericity (ε = 0.702). The results of this ANOVA revealed a significant main effect of condition (F (2.11; 29.5) = 4.138, p = 0.024, η_p_^2^ = 0.228), but not other significant effects for sex (F(1,14) = 1.840, p = 0.196) or for the condition x sex interaction (F(2.11; 29.5) = 0.169, p = 0.855). Follow-up paired comparisons conducted using the dependent t-test yielded, besides the significant results reported in the manuscript, the following non-significant results: MI vs. CI: *t(*15) = 1.496, p = 0.186 (uncorrected p = 0.155); CI vs. BI: *t*(15) = 2.335, p = 0.051 (uncorrected p = 0.034); BI vs. CR: *t*(15) = -0.608, p = 0.552 (uncorrected p = 0.552).

**3.2.2. The effects of condition on the frequency of smiling bouts during the experimental conditions**

Shapiro-Wilk tests indicated that the normality assumption was met: W_MI_ = 0.936, p = 0.308; W_CI_ = 0.939, p = 0.333; W_BI_ = 0.948, p = 0.456; W_CR_ = 0.889, p = 0.054. To assess the effects of condition on the frequency of smiling bouts, a one-way repeated measured ANOVA was conducted with condition as the within-subjects factor. The sphericity assumption was met (*χ^2^*(5) = 7.809; p = 0.168), but the results of this test were non-significant: F(3,45) = 1.360, p = 0.267. A supplementary mixed-model ANOVA was also conducted, with condition as the within-subjects factor and sex as the between-subjects factor. Mauchly's Test of Sphericity further showed that the sphericity assumption was met: *χ^2^*(5)= 7.963; p = 0.159. The results of the ANOVA, however, were non-significant - main effect of condition: F(3,42) = 0.815, p = 0.493; main effect of sex: F(1,14) = 0.024, p = 0.878; interaction effect between condition and sex: F(3,42) = 0.622, p = 0.604.

**3.2.3. The effects of condition on the average duration of smiling bouts during the experimental conditions**

Shapiro-Wilk tests indicated that the normality assumption was violated for the CI and CR conditions: W_MI_ = 0.947, p = 0.441; W_CI_ = 0.687, p < 0.001; W_BI_ = 0.890, p = 0.057; W_CR_ = 0.774, p = 0.001. Logarithmic transformation improved data normality: W_MI_ = 0.945, p = 0.410; W_CI_ = 0.893, p = 0.062; W_BI_ = 0.936, p = 0.306; W_CR_ = 0.920, p = 0.172. A repeated-measures ANOVA with condition as the within-subjects factor. As reported in the manuscript, this test revealed a significant effect of condition (F(3,45) = 4.645, p = 0.007, η_p_^2^ = 0.236).

A supplementary mixed-model ANOVA was also conducted, with condition as the within-subjects factor and sex as a between-subjects factor. Mauchly's Test of Sphericity further showed that the sphericity assumption was met: *χ^2^*(5)= 6.875; p = 0.231. The results of the ANOVA revealed a significant main effect of condition: F(3,42) = 4.419, p = 0.009, η_p_^2^ = 0.240. All other results were non-significant - main effect of sex: F(1,14) = 3.896, p = 0.068; interaction effect between condition and sex: F(3,42) = 0.212, p = 0.888. The follow-up t-tests revealed, besides the significant results reported in the manuscript, the following non-significant results: MI vs. CI: *t(*15) = 1.567, p = 0.166 (uncorrected p = 0.138); CI vs. BI: *t*(15) = 1.939, p = 0.144 (uncorrected p = 0.072); CI vs. CR: *t*(15) = 1.645, p = 0.166 (uncorrected p = 0.121); BI vs. CR: *t*(15) = -0.938, p = 0.363 (uncorrected p = 0.363).

**3.3. THE EFFECTS OF CONDITION ON APPROACH DURING THE EXPERIMENTAL CONDITIONS**

**3.3.1. The effects of condition on approach duration during the experimental conditions**

Shapiro-Wilk tests indicated that the normality assumption was violated for all conditions: W_MI_ = 0.828, p = 0.007; W_CI_ = 0.851, p = 0.014; W_BI_ = 0.706, p < 0.001; W_CR_ = 0.600, p < 0.001. Since normality was not improved by data transformation, a Friedman’s ANOVA was conducted to assess the effects of condition on approach duration. As reported in the manuscript, this test yielded significant results: *χ^2^*(3) = 8.511, p = 0.034. Follow-up paired comparisons using the Wilcoxon Signed-Rank test yielded, besides the significant results reported in the manuscript, the following non-significant results: MI vs. CI: Z = -0.282, p = 0.808 (uncorrected p = 0.808); MI vs. BI: Z = -0.384, p = 0.808 (uncorrected p = 0.735); CI vs. BI: Z=-0.534, p = 0.808 (uncorrected p = 0.626); BI vs. CR: Z = -2.132, p = 0.066 (uncorrected p = 0.033).

**3.3.2.** **The effects of condition on approach frequency during the experimental conditions**

Shapiro-Wilk tests indicated that the normality assumption was violated for three of the four conditions: W_MI_ = 0.919, p = 0.162; W_CI_ = 0.878, p = 0.036; W_BI_ = 0.776, p = 0.001; W_CR_ = 0.730, p < 0.001. Since normality was not improved by data transformation, a Friedman’s ANOVA was conducted to assess the effects of condition on approach frequency. As reported in the manuscript, this test yielded significant results: *χ^2^*(3) = 9.585, p=0.019. Follow-up paired comparisons using the Wilcoxon Signed-Rank test yielded, besides the significant results reported in the manuscript, the following non-significant results: MI vs. CI: Z = -159, p = 0.899 (uncorrected p = 0.899); MI vs. BI: Z = -1.196, p = 0.410 (uncorrected p = 0.273); CI vs. BI: Z = -0.810, p = 0.541 (uncorrected p = 0.451); Bi vs CR: Z = -1.215, p = 0.410 (uncorrected p = 0.245).

**3.3.3. The effects of condition on the average duration of approach bouts during the experimental conditions**

Shapiro-Wilk tests indicated that the normality assumption was violated for three of the four conditions: W_MI_ = 0.779, p = 0.001; W_CI_ = 0.894, p = 0.064; W_BI_ = 0.795, p = 0.002; W_CR_ = 0.852, p = 0.014. Normality was not improved by data transformation, and thus a Friedman’s ANOVA was conducted to assess the effects of condition the average duration of approach bouts. The result of this test was non-significant: *χ^2^*(3) = 5.578, p=0.134.

**3.4. THE EFFECTS OF CONDITION ON TESTING**

**3.4.1 The effects of condition on the overall duration of testing behaviours**

Shapiro-Wilk tests indicated that the normality assumption was violated for two of the four conditions: W_MI_ = 0.960, p = 0.667; W_CI_ = 0.942, p = 0.375; W_BI_ = 0.806, p = 0.003; W_CR_ = 0.666, p < 0.001. As normality was not improved by data transformation, a Friedman’s ANOVA was conducted to assess the effects of condition on testing duration. As reported in the manuscript, the result of this test was significant: *χ^2^*(3) =24.890, p < 0.001. Follow-up paired comparisons using the Wilcoxon Signed-Rank test yielded, besides the significant results reported in the manuscript, the following non-significant results: MI vs. CI: Z = -0.982, p = 0.418 (uncorrected p = 0.348); MI vs. BI: Z = -1.704, p = 0.143 (uncorrected p = 0.095); CI vs. BI: Z = -0.847, p = 0.426 (uncorrected p = 0.426).

**3.4.2. The effects of condition on the frequency of testing bouts**

Shapiro-Wilk tests indicated that the normality assumption was violated for all four conditions: W_MI_ = 0.765, p = 0.001; W_CI_ = 0.870, p = 0.027; W_BI_ = 0.811, p = 0.004; W_CR_ = 0.644, p < 0.001. As normality was not improved by data transformation, a Friedman’s ANOVA was conducted to assess the effects of condition on the frequency of testing bouts. As reported in the manuscript, the result of this test was significant: *χ^2^*(3) = 18.044, p < 0.001.

Follow-up paired comparisons using the Wilcoxon Signed-Rank test yielded, besides the significant results reported in the manuscript, the following non-significant results: MI vs. CI: Z = -0.120, p = 0.908 (uncorrected p = 0.908); MI vs. BI: Z = -1.630, p = 0.137 (uncorrected p = 0.110); CI vs. BI: Z = -1.624, p = 0.137 (uncorrected p = 0.114).

**3.4.3. The effects of condition on the latency to the first testing bout**

Shapiro-Wilk tests indicated that the normality assumption was violated for all four conditions: W_MI_ = 0.727, p < 0.001; W_CI_ = 0.718, p < 0.001; W_BI_ = 0.767, p = 0.001; W_CR_ = 0.611, p < 0.001. As normality was not improved by data transformation, a Friedman’s ANOVA was conducted to assess the effects of condition on latency to the first testing bout. As reported in the manuscript, the result of this test was significant: *χ^2^*(3) =18.600, p < 0.001. Follow-up paired comparisons using the Wilcoxon Signed-Rank test yielded, besides the significant results reported in the manuscript, the following non-significant results: MI vs. CI: Z = -0.776, p = 0.456 (uncorrected p = 0.456); CI vs. BI: Z = -1.790, p = 0.091 (uncorrected p = 0.076).

**3.4.4. Proportion of children that exhibited testing behaviours in each condition**

As reported in the manuscript, a Cochran’s Q test was conducted to determine if the proportion of infants that exhibited testing behaviours differed between the four conditions, and yielded significant results: *χ^2^* (3) = 15.333, *p* = 0.002. The follow-up comparisons using the McNemar test yielded, besides the significant results reported in the manuscript, the following non-significant results: MI vs. CI: p = 0.625 (uncorrected p = 0.625); MI vs. BI: p = 0.126 (uncorrected p = 0.063); CI vs. BI: p = 0.450 (uncorrected p = 0.375); BI vs. CR: p = 0.329 (uncorrected p = 0.219).

**4. EFFECTS OF ORDER ON THE DEPENDENT VARIABLES DURING THE STILL-FACE (SF) INTERVALS - NON-SIGNIFICANT RESULTS**

To determine the effects of order on the dependent variables measured for the SF intervals*,* we used the same approach as in section **1.** above. More specifically, one-way repeated-measures ANOVAs were conducted with SF order as a within-subjects factor. Whenever parametric test assumptions were violated, and data transformation did not improve normality, non-parametric ANOVAs were instead conducted. Whenever necessary, relevant follow-up tests are conducted. To control for false discovery rates (Type I error), p-values for multiple comparisons were corrected using the Benjamini-Hochberg procedure. P-value corrections, however, were not carried out when all p-values a set of multiple comparisons were non-significant. Given the small sample, the Shapiro-Wilk test was employed to determine if data differed significantly from a normal distribution. For the same reason, exact significance (two-tailed) is always reported for the non-parametric tests. Sex was added as a between-subjects factor in order to assess potential interaction effects between condition and sex on the dependent variables.

**4.1. THE EFFECTS OF ORDER ON ATTENTION DURING THE SF INTERVALS**

**4.1.1. Effects of order on the duration of attention during the SF intervals**

Shapiro-Wilk tests showed that the normality assumption was met for all datasets: W_SF1_ = 0.947, p = 0.448; W_SF2_ = 0.951, p = 0.500; W_SF3_ = 0.923, p = 0.191; W_SF4_ = 0.931, p = 0.257. A repeated-measures ANOVA was thus conducted to assess the effects of order on the duration of attention during the SF-intervals. Mauchly’s test indicated that the sphericity assumption was met: *χ^2^*(5) = 4.265; p = 0.513. As reported in the Results section of the manuscript, the results of this test were significant: F(3,45) = 10.232, p < 0.001, η²_p_ = 0.406. Follow-up analyses conducted using the paired t-test revealed, besides the significant differences reported in the manuscript, the following non-significant results: SF-2 vs. SF-3: *t*(15) = 0.245, p = 0.810 (uncorrected p = 0.810); SF-2 vs. SF-4: *t*(15) = 1.112, p = 0.430 (uncorrected p = 0.284); SF-3 vs. SF-4: *t*(15) = 0.684, p = 0.605 (uncorrected p = 0.504). A supplementary mix-model ANOVA was also conducted, with order as the within-subjects factor and sex as the between-subjects factor. Mauchly’s test showed preliminarily that the sphericity assumption was met: *χ^2^*(5) = 2.740; p = 0.741. The results of the mix-model ANOVA did not reveal any additional significant effects besides the effect of order. As such, besides the significant main effect of order (F (3,42) = 13.179, p < 0.001, η_p_² = 0.485), there was a non-significant main effect of sex (F (1,14) = 3.352, p = 0.088), and a non-significant interaction effect between sex and order (F (3,42) = 2.529, p = 0.070.

**4.1.2. Effects of order on the frequency of attention bouts during the SF-phase**

Shapiro-Wilk tests revealed that the normality assumption was violated by the SF-1 and SF-4 datasets: W_SF1_ = 0.881, p = 0.034; W_SF2_ = 0.923, p = 0.166; W_SF3_ = 0.916, p = 0.126; W_SF4_ = 0..829, p = 0.005. Data transformation could not sufficiently improve normality, and thus a Friedman’s ANOVA was conducted to assess the effects of order on the frequency of attention bouts during the SF-intervals. The results of this test were non-significant (*χ^2^*(3) = 6.432, p = 0.091), and thus no subsequent follow-up comparisons were conducted.

**4.1.3.** **Effects of order on average duration of attention bouts during the SF-intervals**

Shapiro-Wilk tests showed that the normality assumption was met for all datasets: W_SF1_ = 0.901, p = 0.084; W_SF2_ = 0.903, p = 0.090; W_SF3_ = 0.980, p = 0.960; W_SF4_ = 0.967, p = 0.665. A repeated-measures ANOVA was thus conducted to assess the effects of order on the average duration of attention bouts during the SF intervals. Mauchly’s test showed that the sphericity assumption was met: *χ^2^*(5) = 3.467, p = 0.871. As reported in the manuscript, the results of this ANOVA were significant: F (3.45) =6.602, p = 0.001, η_p_² = 0.306. Follow-up comparisons based on the paired t-test revealed, besides the significant results reported in the manuscript, the following non-significant results: SF-2 vs. SF-3: *t*(15) = 0.835, p = 0.500 (uncorrected p = 0.417), SF-2 vs. SF-4: *t*(15) = 0.883, p = 0.587 (uncorrected p = 0.391), SF-3 vs. SF-4: *t*(15) = -0.066, p = 0.948 (uncorrected p = 0.948). A complementary mix-model ANOVA, with order as the within-subjects factor and sex as a between-subjects factor was conducted. Mauchly’s test revealed that the sphericity assumption was met: *χ^2^*(5) = 3.301, p = 0.655. Besides the significant effect of order (reported in the manuscript: F(3,42) = 5.808, p = 0.002, η²_p_ = 0.293), there was a non-significant main effect of sex (F(1,14) = 1.916, p = 0.188), and a non-significant interaction effect of order and sex (F (3,42) = 0.213, p = 0.887).

**4.2. SMILING**

**4.2.1. Effects of order on overall duration of smiling during the SF intervals**

Shapiro-Wilk tests revealed that the normality assumption was severely violated for all: W_SF1_ = 0.848, p = 0.013; W_SF2_ = 0.723, p < 0.001; W_SF3_ = 0.670, p < 0.001; W_SF4_ = 0.352, p < 0.001. Data transformation did not sufficiently improve normality, and thus a Friedman’s ANOVA was conducted to assess the effects of order on the overall duration of smiling during the SF intervals. As reported in the manuscript, the results of this test were significant: *χ^2^*(3) = 13.703, p = 0.002. Follow-up comparisons using the Wilcoxon Signed-Rank test yielded, besides the significant results reported in the manuscript, the following non-significant results: SF-2 vs. SF-3: Z = -0.889, p = 0.413 (uncorrected p = 0.413); SF-2 vs. SF-4: Z = -1.836, p = 0.111 (uncorrected p = 0.074); SF-3 vs. SF-4: Z = -1.400, p = 0.234 (uncorrected p = 0.195).

**4.2.2. Effects of order on the frequency of smiling bouts during the SF intervals**

Shapiro-Wilk tests showed that the normality assumption was violated for all data sets. As such, W_SF1_ = 0.857, p = 0.018; W_SF2_ = 0.697, p < 0.001; W_SF3_ = 0.751, p = 0.001; W_SF4_ = 0.398, p < 0.001. Data transformation could not sufficiently improve normality, and thus a Friedman’s ANOVA was conducted to assess the effects of order on the frequency of smiling bouts. As reported in the manuscript, the results of this test were significant: *χ^2^*(3) = 13.212, p = 0.002. Follow-up comparisons using the Wilcoxon Signed-Rank test were conducted, which yielded, besides the significant results reported in the manuscript, the following non-significant results: SF-1 vs. SF-2: Z = -1.222, p = 0.366 (uncorrected p = 0.305); SF-1 vs. SF-3: Z = -1.910, p = 0.138 (uncorrected p = 0.069); SF-2 vs. SF-3: Z = -1.026, p = 0.408 (uncorrected p = 0.408); SF-2 vs. SF-4: Z = -2.154, p = 0.141 (uncorrected p = 0.047); SF-3 vs. SF-4: Z = -1.983, p = 0.117 (uncorrected p = 0.078).

**4.2.3. Effects of order on the average duration of smiling bouts during the SF intervals**

Shapiro-Wilk tests revealed that the normality assumption was violated for three datasets: W_SF1_ = 0.890, p = 0.056; W_SF2_ = 0.750, p = 0.001; W_SF3_ = 0.767, p = 0.001; W_SF4_ = 0.352, p < 0.001. As data transformation did not improve normality, the effects of order on the average duration of smiling bouts during the SF intervals was assessed by conducting a Friedman’s ANOVA. As reported in the manuscript, the results of this test were significant: *χ^2^*(3) = 14.838, p = 0.001. Follow-up comparisons based on the Wilcoxon Signed-Rank test yielded, besides the significant results reported in the manuscript, the following non-significant results: SF-1 vs. SF-3: Z = -2.201, p = 0.054 (uncorrected p = 0.027); SF-2 vs. SF-3: Z = -0.445, p = 0.840 (uncorrected p = 0.700); SF-2 vs. SF-4: Z = -1.836, p = 0.740 (uncorrected p = 0.074), and SF-3 vs. SF-4: Z = -1.260, p = 0.375 (uncorrected p = 0.250).

**4.3. APPROACH**

**4.3.1. Effects of order on overall duration of approach during the SF intervals**

Shapiro-Wilk tests revealed that the normality assumption was severely violated for all datasets: W_SF1_ = 0.512, p < 0.001; W_SF2_ = 0.569, p < 0.001; W_SF3_ = 0.598, p < 0.001; W_SF4_ = 0.403, p < 0.001. Data transformation did not improve normality, and thus a Friedman’s ANOVA was conducted in order to assess the effects of order on the overall duration of approach behaviours during the SF intervals. The results of this test were non-significant (*χ^2^*(3) = 0.452, p = 0.939), and thus no follow-up comparisons were conducted.

**4.3.2. Effects of order on the frequency of approach bouts during the SF intervals**

Shapiro-Wilk tests revealed that the normality assumption was severely violated for all datasets: W_SF1_= 0.554, p < 0.001; W_SF2_ = 0.587, p < 0.001; W_SF3_ = 0.546, p < 0.001; W_SF4_ = 0.398, p < 0.001. As normality could not be improved by data transformation, a Friedman’s ANOVA was conducted to assess the effects of order on the frequency of approach bouts during the SF intervals. The results of this test were non-significant (*χ^2^*(3) = 1.329, p = 0.753), and thus no follow-up comparisons were conducted.

**4.3.3. Effects of order on the average duration of approach bouts during the SF intervals**

Shapiro-Wilk tests showed that the normality assumption was severely violated for all datasets: W_SF1_ = 0.657, p < 0.001; W_SF2_ = 0.594, p < 0.001; W_SF3_ = 0.598, p < 0.001; W_SF4_ = 0.403, p < 0.001. Since data normality could not be improved by data transformation, the effects of order on the average duration of approach bouts during the SF intervals was assessed by conducting a Friedman’s ANOVA. As the results of this test were non-significant (*χ^2^*(3) = 1.027, p = 0.820), no follow-up comparisons were conducted.

**4.4. SOCIAL BIDDING**

**4.4.1. Effects of order on the overall duration of social bidding during the SF intervals**

Shapiro-Wilks tests indicated that the normality assumption was violated for all data sets: W_SF1_ = 0.875, p = 0.033; W_SF2_ = 0.839, p 0.009; W_SF3_ = 0.782, p = 0.002; W_SF4_ = 0.801, p = 0.003. Since normality was not sufficiently improved by data transformation, a Friedman’s ANOVA was conducted in order to assess the effects of order on the overall duration of social bidding during the SF intervals. The results of this test were non-significant: *χ^2^*(3) = 5.779, p = 0.122.

**4.4.2. Effects of order on the frequency of social bidding during the SF intervals**

Shapiro-Wilks tests showed that the normality assumption was violated for two data sets: W_SF1_ = 0.837, p = 0.009; W_SF2_ = 0.927, p = 0.218; W_SF3_ = 0.887, p = 0.050; W_SF4_ = 0.808, p = 0.003. Since normality was not improved by data transformation, a Friedman’s ANOVA was conducted to assess the effects of order on the frequency of social bidding during the SF intervals. As reported in the manuscript, this test yielded significant results: *χ^2^*(3) = 11.167, p = 0.008. Follow-up comparisons using the Wilcoxon Sign-rank test yielded, besides the significant results reported in the manuscript, the following non-significant results : SF1 vs. SF2: Z = -1.355, p = 0.255 (uncorrected p = 0.212); SF1 vs. SF3 = -1.568, p = 0.274 (uncorrected p = 0.137); SF2 vs. SF 3 = -0.733, p = 0.523 (uncorrected p = 0.523); SF 2 vs. SF 4: Z = -2.167, p = 0.087 (uncorrected p = 0.029), SF 3 vs. SF 4 = -1.496, p = 0.270 (uncorrected p = 0.180).

**4.4.3. Effects of order on the average duration of social bidding bouts during the SF intervals**

Shapiro-Wilks tests indicated that the normality assumption was violated for two data sets: W_SF1_ = 0.922, p = 0.184; W_SF2_ = 0.916, p = 0.143; W_SF3_ = 0.782, p = 0.002; W_SF4_ = 0.814, p = 0.004. Since normality was not sufficiently improved by data transformation, a Friedman’s ANOVA was conducted in order to assess the effects of order on the overall duration of social bidding during the SF intervals. The results of this test were non-significant: *χ^2^*(3) = 3.359, p = 0.346

**5. EFFECTS OF SEX ON THE DEPENDENT VARIABLES DURING THE STILL-FACE (SF) intervals - NON-SIGNIFICANT RESULTS**

To assess the effects of sex on the 12 dependent variables, a similar approach to that used in section **2.** above was used. More specifically, independent t-test or Mann-Whitney U tests were conducted depending on whether the data met parametric test assumptions. To further capture potential interaction effects between sex and order, or between sex and condition, additional tests (independent t-tests or Mann-Whitney U tests) were conducted separately for each experimental slot and each experimental condition, with sex as the grouping variable.

**5.1. THE EFFECTS OF SEX ON ATTENTION DURING THE SF intervals**

**5.1.1. The effects of sex on the total duration of attention during the SF intervals**

Shapiro-Wilk tests revealed that the normality assumption was violated for both datasets:

W_Males_ = 0.932, p = 0.013; W_Females_ = 0.886, p = 0.023. While logarithmic transformation could not sufficiently improve data normality, square root transformation did: W_Males_ = 0.978, p = 0.553; W_Females_ = 0.969, p = 0.728. An independent t-test was thus conducted on square root transformed data in order to assess the effects of sex on the overall duration of attention during the SF intervals. As reported in the manuscript, the results of this test were significant (*t*(14) = 2.204, p = 0.031, *d* = 0.594). The effect of sex on the total duration of attention was further assessed separately, for each order slot and for each condition. Besides the two interaction effects reported in the main manuscript, the results of these tests were all non-significant. As such with respect to order, for SF-2 *t*(14) = 0.338, p = 0.741; for SF-3, *t*(14) = 0.289, p = 0.777, and for SF-4 *t*(14) = 1.990, p = 1.073. With respect to condition, for the SF following the MI condition: *t*(14) = -.289, p = 0.777; for the SF following the BI condition: *t*(14) = - 0.786, p = 0.445; for the SF following the CR condition: *t*(14) = -0.279, p = 0.784.

**5.1.2. The effects of sex on the frequency of attention bouts during the SF intervals**

Shapiro-Wilk tests revealed that the normality assumption was violated for the male group: W_Males_ = 0.899, p = 0.001; W_Females_ = 0.932, p = 0.167. Since normality was not improved by data transformation, a Mann-Whitney test was conducted to assess the effects of sex on the frequency of attention bouts. This test yielded non-significant results: U = 330.000, p = 0.104. Separate tests were further conducted in order to assess the effect of sex for the each SF-phase as a function of condition and order. All these tests yielded non-significant results: U_SF-MI_ = 20.000, p = 0.441; U_SF-CI_ = 13.500, p = 0.115; U_SF-BI_ = 24.500, p = 0.743; U_SF-CR_ = 24.500, p = 0.743; U_SF-1_ = 18.500, p = 0.716 (uncorrected p = 0.358); U_SF-2_ = 23.000, p = 0.9 (uncorrected p = 0.675); U_SF-3_ = 25.500, p = 0.875 (uncorrected p = 0.875); U_SF-4_ = 10.500 p = 0.172 (uncorrected p = 0.043).

**5.1.3. The effects of sex on the average duration of attention bouts during the SF intervals**

Shapiro-Wilk tests indicated that the normality assumption was violated for the male group: W_Males_ = 0.923, p = 0006; W_Females_ = 0.911, p = 0.067. Logarithmic transformation improved data normality (W_Males_ = 0.968, p = 0.252; W_Females_ = 0.981, p = 0.948) and thus, in order to assess the effects of sex on the average duration of attention bouts, an independent t-test was conducted on log transformed data. The results of this test were non-significant: *t*(14) = -1.648, p = 0.104. Additional tests were conducted to separately assess the effects of sex for each SF phase as a function of condition and order. The results of all these tests were non-significant: U_SF-MI_ = 26.000, p = 0.913; U_SF-CI_ = 11.000, p = 0.069; U_SF-BI_ = 24.000, p = 0.743; U_SF-CR_ = 26.000, p = 0.913; for SF-1: *t*(14) = -0.969, p =0.349; for SF-2: *t*(14) = -0.296, p = 0.771; for SF-3: *t*(14) = -1.619, p = 0.128; for SF-4: *t*(14) = -0.932, p = 0.367. Note that the latter t-tests were conducted on log transformed data.

**5.2. THE EFFECTS OF SEX ON SMILING DURING THE SF intervals**

**5.2.1. The effects of sex on the total duration of smiling in the SF intervals**

The Shapiro-Wilk test revealed that the normality assumption was severely violated for both datasets: W_Males_ = 0.686, p < 0001; W_Females_ = 0.618, p < 0001. Since data transformation did not improve normality, athe Mann-Whitney test was used to assess the effects of sex on the overall duration of smiling during the SF-intervals. The test yielded non-significant results: U = 385.500, p = 0.389. Additional tests were conducted to assess the effects of sex separately for each SF-phase, as a function of condition and order. All these tests yielded non-significant results: U_SF-MI_ = 25.000, p = 0.924; U_SF-CI_ = 25.500, p = .846; U_SF-BI_ = 25.500, p = 0.846; U_SF-CR_ = 15.000, p = 0.128; U_SF-1_ = 26.000, p = 0.911; U_SF-2_ = 27.000, p = 0.974; U_SF-3_ = 21.000, p = 0.492; U_SF-4_ = 22.500, p = 0.542.

**5.2.2. The effects of sex on the frequency of smiling bouts during the SF intervals**

Shapiro-Wilk tests revealed that the normality assumption was severely violated for both datasets: W_Males_ = 0.705, p < 0001; W_Females_ = 0.667, p < 0001. As normality was not improved by data transformation, a Mann-Whitney U test was conducted to assess the effects of sex on the frequency of smiling bouts during the SF intervals. The test yielded non-significant results: U = 412.500, p = 0.663. Further analyses were conducted to assess the effects of sex on smiling frequency in each SF phase, as a function of condition and order. The results of these tests were non-significant: U_SF-MI_ = 24.000, p = 0.811; U_SF-CI_ = 27.000, p = 1.000; U_SF-BI_ = 26.500, p = 0.923; U_SF-CR_ = 16.500, p = 0.227; U_SF-1_ = 25.500, p = 0.810; U_SF-2_ = 24.000, p = 0.705; U_SF-3_ = 21.000, p = 0.464; U_SF-4_ = 22.500, p = 0.542.

**5.2.3. The effects of sex on the average duration of smiling bouts during the SF intervals**

Shapiro-Wilk tests revealed that the normality assumption was severely violated for both datasets: W_Males_ = 0.754, p < 0001; W_Females_ = 0.670, p < 0001. Normality could not be improved by data transformation, and thus a Mann-Whitney U test was conducted to assess the effects of sex on the average duration of smiling bouts during the SF intervals. This test yielded non-significant results: U = 357.500, p = 0.191. The effects of sex on the average duration of smiling bouts was further assessed separately, for each SF phase, as a function of condition and order. All these tests yielded non-significant results: U_SF-MI_ = 22.500, p = 0.840; U_SF-CI_ = 21.500, p = 0.674; U_SF-BI_ = 14.000, p = 0.179; U_SF-CR_ = 12.000, p = 0.089; U_SF-1_ = 19.000, p = 0.370; U_SF-2_ = 24.000, p = 0.712; U_SF-3_ = 21.000, p = 0.492; U_SF-4_ = 22.500, p = 0.542.

**5.3. THE EFFECTS OF SEX ON APPROACH BEHAVIOURS**

**5.3.1. The effects of sex on the total duration of approach behaviours during the SF intervals**

Shapiro-Wilk tests revealed that the normality assumption was severely violated for both datasets: W_Males_ = 0.436, p < 0001; W_Females_ = 0.601, p < 0001. Since data transformation did not improve normality, a Mann-Whitney U test was conducted to assess the effects of sex on the overall duration of approach behaviours during the SF intervals. This test yielded non-significant results: U = 403.000, p = 0.487. The effects of sex were further assessed for each SF phase separately, as a function of condition and order. All these tests yielded non-significant results: U_SF-MI_ = 23.000, p = 0.622; U_SF-CI_ = 20.000, p = 0.509; U_SF-BI_ = 23.000, p = 0.622; U_SF-CR_ = 25.000, p = 1.000; U_SF-1_ = 27.000, p = 1.000 (uncorrected p = 1.000); U_SF-2_ = 17.500, p = 0.490 (uncorrected p = 0.245); U_SF-3_ = 12.000 , p = 0.072 (uncorrected p = 0.018); U_SF-4_ = 25.000, p = 1.000 (uncorrected p = 1.000).

**5.3.2. The effects of sex on the frequency of approach bouts during the SF intervals**

Shapiro-Wilk tests revealed that the normality assumption was severely violated for both datasets: W_Males_ = 0.500, p < 0001; W_Females_ = 0.632, p < 0001. As normality was not improved by data transformation, a Mann-Whitney U test was conducted to assess the effects of sex on the frequency of approach bouts during the SF intervals. The test yielded negative results: U = 406.000, p = 0.519. Further analyses were conducted to reveal interaction effects by assessing the effects of sex on the frequency of approach bouts in each SF phase, as a function of condition and order. The results of these tests were non-significant: U_SF-MI_ = 23.500, p = 0.736; U_SF-CI_ = 19.000, p = 0.214; U_SF-BI_ = 22.000, p = 0.509; U_SF-CR_ = 25.000, p = 1.000; U_SF-1_ = 26.000, p = 1.000; U_SF-2_ = 17.500, p = 0.245; U_SF-3_ = 13.500, p = 0.063; U_SF-4_ = 24.500, p = 1.000.

**5.3.3. The effects of sex on the average duration of approach bouts during the SF intervals**

Shapiro-Wilk tests revealed that the normality assumption was severely violated for both datasets: W_Males_ = 0.754, p < 0001; W_Females_ = 0.616, p < 0.001. Normality could not be improved by data transformation, and thus a Mann-Whitney U test was conducted to assess the effects of sex on the average duration of approach bouts during the SF intervals. This test yielded non-significant results: U = 396.000, p = 0.418. The effects of sex on the average duration of approach bouts was further assessed separately, for each SF phase, as a function of condition and order. All these tests yielded non-significant results: U_SF-MI_ = 23.000, p = 0.622; U_SF-CI_ = 20.000, p = 0.509; U_SF-BI_ = 24.000, p = 0.773; U_SF-CR_ = 24.000, p = 0.771; U_SF-1_ = 26.000, p = 1.000; U_SF-2_ = 17.500, p = 0.245; U_SF-3_ = 13.500, p = 0.063; U_SF-4_ = 24.500, p = 1.000.

**5.4. THE EFFECTS OF SEX ON SOCIAL BIDDING**

**5.4.1.** **The effects of sex on the overall duration of social bidding during the SF intervals**

Shapiro-Wilk tests revealed that the normality assumption was violated for both datasets: W_Males_ = 0.806, p < 0001; W_Females_ = 0.897, p = 0.036. Since data normality could not be improved by data transformation, a Mann-Whitney U test was conducted to assess the effects of sex on the overall duration of social bidding during the SF intervals. The results of this test were non-significant: U = 340.000, p = 0.146. Additional data analyses using the Mann-Whitney U test were conducted separately as a function of order and condition, and all these tests yielded non-significant results suggesting the absence of interaction effects between sex and order, or sex and condition. As such, U_SF-MI_ = 22.000, p = 0.559; U_SF-CI_ = 11.000, p = 0.064; U_SF-BI_ = 23.000, p = 0.660; U_SF-CR_ = 14.000, p = 0.138; U_SF-1_ = 13.000, p = 0.115; U_SF-2_ = 26.000, p = 0.913; U_SF-3_ = 18.500, p = 0.329; U_SF-4_ = 25.000, p = 0.803.

**5.4.2.** **The effects of sex on the frequency of social bidding during the SF intervals**

Shapiro-Wilk tests revealed that the normality assumption was severely violated for both datasets: W_Males_ = 0.875, p < 0001; W_Females_ = 0.837, p = 0.003. Data normality was not improved by data transformation, and thus a Mann-Whitney U test was conducted to assess the effects of sex on the overall duration of social bidding during the SF intervals, which yielded non-significant results: U = 411.500, p = 0.676. To assess potential interaction effects between sex and order, or sex and condition, additional data analyses using the Mann-Whitney U test were conducted separately as a function of order and condition. The results of all these tests were non-significant. As such, U_SF-MI_ = 21.500, p = 0.497; U_SF-CI_ = 18.500, p = 0.325; U_SF-BI_ = 26.500, p = 1.000; U_SF-CR_ = 23.000, p = 0.659; U_SF-1_ = 19.500, p = 0.406; U_SF-2_ = 22.500, p = 0.625; U_SF-3_ = 27.000, p = 1.000; U_SF-4_ = 24.500, p = 0.773.

**5.4.3.** **The effects of sex on the average duration of social bidding during the SF intervals**

Shapiro-Wilk tests revealed that the normality assumption was severely violated for the male group: W_Males_ = 0.860, p < 0001; W_Females_ = 0.941, p = 0.254. Data normality was sufficiently improved by logarithmic transformation (W_Males_ = 0.967, p = 0.433; W_Females_ = 0.963, p = 0.724), and thus an independent t-test was conducted to assess the effects of sex on the average duration of social bidding bouts during the SF intervals. The results of this test were non-significant: *t*(62) = -1.713, p = 0.092. Additional data analyses were conducted to capture potential interaction effects between sex x condition, or between sex x order. Shapiro-Wilks test indicated that the normality assumption was violated for several datasets. Data normality was improved by logarithmic transformation for the sex x condition datasets, and independent t-tests were run to assess the potential interaction effects between sex x condition on the average duration of social bidding bouts during the SF intervals. The results of all these tests were non-significant. As such, for SF-MI, *t*(14) = 0.963, p = 0.352, for SF-CI, *t*(14) = -1.609, p = 0.130; for SF-BI: *t*(14) = -0.625, p = 0.542; SF-CR: *t*(14) = -2.015, p = 0.064. All the Mann-Whitney U tests conducted to assess interaction effects between sex x order yielded non-significant results: U_SF-1_ = 27.000, p = 1.000 (uncorrected p = 1.000); U_SF-2_ = 17.500, p = 0.490 (uncorrected p = 0.245); U_SF-3_ = 12.000, p = 0.072 (uncorrected p = 0.018); U_SF-4_ = 25.000, p = 1.000 (uncorrected p = 0.001).

**6. THE EFFECTS OF CONDITION ON THE DEPENDENT VARIABLES DURING THE STILL-FACE (SF) INTERVALS - NON-SIGNIFICANT RESULTS**

To determine the effects of condition on the dependent variables during the SF intervals, we used a similar approach to that described in section **3.** above. More specifically, to assess the effects of condition on responses during the subsequent SF-intervals, one-way repeated-measures ANOVAs were conducted with condition as a within-subjects factor. Whenever parametric test assumptions were violated, and data transformation did not improve normality, non-parametric ANOVAs were instead conducted. If necessary, relevant follow-up tests are conducted. In addition, to assess interaction effects between condition and sex mixed-model ANOVAs were also conducted with condition as the within-subject factor and sex as a between-subjects factor. To control for false discovery rates (Type I error), p-values for all multiple comparisons were corrected using the Benjamini-Hochberg procedure. P-value corrections, however, were not carried out when all p-values in each set of multiple comparisons were non-significant. Given the small sample, the Shapiro-Wilk test was employed to determine if data differed significantly from a normal distribution. For the same reason, exact significance (two-tailed) is always reported for the non-parametric tests.

**6.1. THE EFFECTS OF CONDITION ON ATTENTION DURING THE SF INTERVALS**

**6.1.1. Effects of condition of the overall duration of attention in the SF intervals**

Shapiro-Wilk tests indicated that the normality assumption was met for all datasets. As such, W_SF-MI_ = 0.938, p = 0.326; W_SF-CI_ = 0.892, p = 0.060; W_SF-BI_ = 0.979, p = 0.956; W_SF-CR_ = 0.922, p = 0.181. Mauchly’s test of sphericity showed that the sphericity assumption was met: *χ*^2^(5)= 6.862; p = 0.232. A repeated-measures ANOVA was thus conducted to assess the effects of condition on the overall duration of attention in the SF intervals, which yielded non-significant results: F(3,45) = 1.428, p = 0.247. Thus, no follow-up focused comparisons were done. In addition, we also conducted a mixed- model ANOVA to assess the effects of condition and sex on the overall duration of attention in the SF intervals. Mauchly's Test of Sphericity showed that the sphericity assumption was also met: *χ^2^*(5) = 4.154; p = 0.528. As reported in the manuscript, this mix-model ANOVA revealed a significant main effect of condition (F(3,42) = 3.193, p = 0.033, η_p_² = 0.186), a non-significant main effect of sex (F(1,14) = 3.352, p = 0.088), and a significant interaction effect between condition and sex: F(3,42) =, 2.938, p = 0.044, η_p_² = 0.173). To follow-up on the effect of condition, follow-up paired comparisons using the paired t-test were conducted, but all results were non-significant. As such, for SF-MI vs. SF-CI: t(15) = -1.053, p = 0.618 (uncorrected p = 0.309); SF-MI vs. SF-BI: t(15) = 0.986, p = 0.510 (uncorrected p = 0.340); SF-MI vs. SF-CR: t(15) = 0.434, p = 0.804 (uncorrected p = 0.670); SF-CI vs. SF-BI: t(15) = 1.835, p = 0.516 (uncorrected p = 0.086); SF-CI vs. SF-CR: t(15) = 1.372, p = 0.570 (uncorrected p = 0.190); SF-BI vs. SF-CR: t(15) = -0.313, p = 0.759 (uncorrected p = 0.759). To follow-up on the significant interaction effect between sex x condition, independent t-tests were conducted separately for each condition dataset, with sex as the between-subjects variable. Besides the interaction effect reported in the manuscript, all the other tests yielded non-significant results: SF-MI: t(14) = 0.289, p = 0.777; SF-BI: t(14) = 0.786, p = 0.445; SF-CR: t(14) = 0.279, p = 0.784.

**6.1.2. Effects of condition on the frequency of attention bouts during the SF intervals**

Shapiro-Wilk tests indicated that the normality assumption was violated for the SF intervals following the CI and CR conditions: W_SF-MI_ = 0.915, p = 0.139; W_SF-CI_ = 0.874, p = 0.032; W_SF-BI_ = 0.929, p = 0.235; W_SF-CR_ = 0.870, p = 0.027. As data transformation did not improve normality, a Friedman’s ANOVA was conducted to assess the effect of condition on the frequency of attention bouts in the subsequent SF intervals. The results of this test were non-significant: *χ^2^*(3) = 3.176, p = 0.374.

**6.1.3. The effects of condition on the average duration of attention bouts during the SF intervals**

Shapiro-Wilk tests indicated that the normality assumption was violated for the SF intervals following the CI and BI conditions: W_SF-MI_ = 0.939, p = 0.338; W_SF-CI_ = 0.884, p = 0.045; W_SF-BI_ = 0.870, p = 0.027; W_SF-CR_ = 0.907, p = 0.102. While logarithmic transformation did not sufficiently improve data normality, a square root transformation did: W_SF-MI_ = 0.961, p = 0.672; W_SF-CI_ = 0.945, p = 0.415; W_SF-BI_ = 0.910, p = 0.117; W_SF-CR_ = 0.939, p = 0.339. To assess the effect of condition on the average bout duration in the SF intervals, a repeated-measures ANOVA was thus conducted on square root transformed data. The sphericity assumption was met, as Mauchly’s test was non-significant: *χ^2^*(5) = 3.698; p = 0.595. The results of the ANOVA were also non-significant: F (3,45) = 1.308, p = 0.283. A complementary mix-model ANOVA was also conducted to assess the effects of sex and condition on the average duration of attention bouts in the SF intervals. Mauchly’s test was non-significant indicating that the sphericity assumption was not violated: *χ^2^*(5) = 4.940; p = 0.424. The ANOVA yielded a non-significant main effect of condition (F (3,42) = 1.930, p = 0.139), a non-significant main effect of sex (F (1,14) = 1.914, p = 0.188), and a non-significant interaction effect of sex x condition (F (3,42) = 1.040, p = 0.385).

**6.2. THE EFFECTS OF CONDITION ON SMILING DURING THE SF INTERVALS**

**6.2.1. The effects of condition on the overall duration of smiling during the SF intervals**

Shapiro-Wilk tests indicated that the normality assumption was severely violated for all data sets: W_SF-MI_ = 0.539, p < 0.001; W_SF-CI_ = 0.695, p < 0.001; W_SF-BI_ = 0.762, p < 0.001; W_SF-CR_ = 0.716, p < 0.001. Data transformation could not sufficiently improve data normality. This was to be expected given the high number of cells with null value. A Friedman’s ANOVA was nevertheless conducted to assess the effects of condition on the duration of smiling in the SF intervals. The results of this test were non-significant: *χ^2^*(3) = 1.811, p = 0.631.

**6.2.2. The effects of condition on the frequency of smiling bouts during the SF intervals**

Shapiro-Wilk tests indicated that the normality assumption was severely violated for all data sets: W_SF-MI_ = 0.573, p < 0.001; W_SF-CI_ = 0.779, p = 0.001; W_SF-BI_ = 0.747, p = 0.001; W_SF-CR_ = 0.714, p < 0.001. Data transformation could not sufficiently improve data normality. This was to be expected given the high number of cells with null value. The results of a Friedman’s ANOVA conducted on the non-transformed data were non-significant: *χ^2^*(3) = 2.273, p = 0.531.

**6.2.3. The effects of condition on the average duration of smiling bouts during the SF intervals**

Shapiro-Wilk tests indicated that the normality assumption was severely violated for all data sets: W_SF-MI_ = 0.617, p < 0.001; W_SF-CI_ = 0.759, p = 0.001; W_SF-BI_ = 0.783, p = 0.002; W_SF-CR_ = 0.670, p < 0.001. prove data normality. This was to be expected given the high number of cells with null value. The results of a Friedman’s ANOVA conducted on the non-transformed data were non-significant: *χ^2^*(3) = 2.676, p = 0.457.

**6.3. THE EFFECTS OF CONDITION ON APPROACH DURING THE SF INTERVALS**

**6.3.1. The effects of condition on approach duration during the SF intervals**

Shapiro-Wilk tests indicated that the normality assumption was severely violated for all data sets: W_SF-MI_ = 0.644, p < 0.001; W_SF-CI_ = 0.494, p < 0.001; W_SF-BI_ = 0.569, p < 0.001; W_SF-CR_ = 0.385, p < 0.001. Data transformation could not sufficiently improve normality, and thus a Friedman’s ANOVA was conducted to assess the effects of condition on the duration of approach during the SF intervals. The results of this test were non-significant: *χ^2^*(3) = 3.575, p = 0.323.

**6.3.2.** **The effects of condition on approach frequency**

Shapiro-Wilk tests indicated that the normality assumption was severely violated for all SF phases: W_SF-MI_ = 0.644, p < 0.001; W_SF-CI_ = 0.484, p < 0.001; W_SF-BI_ = 0.644, p < 0.001; W_SF-CR_ = 0.344, p < 0.001. The high number of null values would make statistical treatment meaningless. A Friedman’s ANOVA test was nevertheless conducted, which yielded a non-significant result: The results of this test were non-significant: *χ^2^*(3) = 2.357, p = 0.521.

**6.3.3. The effects of condition on the average duration of approach bouts during the SF intervals**

The normality assumption was severely violated for all datasets, as indicated by Shapiro-Wilks tests: W_SF-MI_ = 0.643, p < 0.001; W_SF-CI_ = .494, p < 0.001; W_SF-BI_ = .636, p < 0.001; W_SF-CR_ = .409, p < 0.001. As expected, given the high number of cells with null results, data transformation did not improve the normality of distribution. A Friedman’s ANOVA was nonetheless conducted to assess the effect of condition on the average duration of attention bouts during the SF phase. The test yielded non-significant results: *χ^2^*(3) = 3.658, p = 0.312

**6.4. THE EFFECTS OF CONDITION ON SOCIAL BIDDING**

**6.4.1. The effects of condition of the overall duration of social bidding**

Shapiro-Wilks tests indicated that the normality assumption was violated for three of the data sets: W_SF-MI_ = 0.847, p = 0.010; W_SF-CI_ = 0.817, p = 0.004; W_SF-BI_ = 0.876, p = 0.028;

W_SF-CR_ = 0.875, p = 0.027. Since normality was not improved by data transformation, a non-parametric ANOVA was conducted on non-transformed data. The results of this test were non-significant (*χ^2^*(3) = 1.349, p = 0.732), and thus no follow-up paired comparisons were run.

**6.4.2. The effects of condition of the frequency of social bidding during the SF intervals**

The normality assumption was violated for three of the four datasets, as indicated by Shapiro-Wilks tests: W_SF-MI_ = 0.837, p = 0.009; W_SF-CI_ = 0.217, p = 0.043; W_SF-BI_ = 0.732, p < 0.000; W_SF-CR_ = 0.805, p = 0.003. As data transformation did not sufficiently improve normality, a Friedman’s ANOVA was conducted to assess the effects of condition of the frequency of social bidding during the SF intervals. The results of this test were non-significant: *χ^2^*(3) = 2.262, p = 0.532.

**6.4.2. The effects of condition of the average duration of social bidding bouts during the SF intervals**

Shapiro-Wilks tests revealed that the normality assumption was violated for two datasets: W_SF-MI_ = 0.889, p = 0.054; W_SF-CI_ = 0.855, p = 0.016; W_SF-BI_ = 0.918, p = 0.155; W_SF-CR_ = 0.842, p = 0.010. Since data normality could not be improved by logarithmic transformation, a Friedman's ANOVA was conducted in order to assess the effects of condition on the average duration of social bidding bouts during the SF intervals. The results of this test were non-significant: *χ^2^*(3) = 0.148, p = 0.989.
