# Supplementary material for: Imitation recognition and its prosocial effects in 6-month old infants
Source: PLoS One. 2020 May 20;15(5):e0232717. doi: 10.1371/journal.pone.0232717 (PMC7239450; doi:10.1371/journal.pone.0232717)
Supplement: Attachment — Submitted filename: Response to reviewers.docx [file pone.0232717.s009.docx]

Reviewer #1: The manuscript is well written and it sound. It is easy to follow and I think it absolutely deserves to be published on Plos One.

Materials and Methods are accurate and results are shown in a very accurate and clear way.

The only thing that is not so clear is the meaning of the asterisks in the figure. What does one asterisk means? and to asterisks? I think authors should explicit the meaning in the figure legend.

REPLY: We are grateful for this observation. This has been remedied by specifying in each figure caption that * indicates p < 0.005; ** indicates p < 0.01; *** indicates p < 0.001 (lines 629-630, 635-636, 641-642, and 698-699 in the revised manuscript)

Reviewer #2: The authors should provide a more detailed explanation of the following:

1. Methods

Reviewer #2, concern (1):

CR condition - Despite the detailed and comprehensive evaluation of the control conditions used in previous studies on infant imitation recognition and the attempt by the authors to overcome any potential limitations by using a different approach, I have some concern about the CR condition used in the current study. The purpose of a non-imitative contingent control condition in imitation studies is to disentangle the effect of temporal contingency from the one driven by the structural (and temporal) contingency. In fact, in both human and non-human primates, it has been shown that subjects recognize when being imitated only when the action performed by the imitator is both temporally AND structurally contingent as well as attuned in terms of affective valence. In the current study, the authors decided to use a pre-set inventory of 3 actions and presented them every 20 seconds. Thus, the experimenter performed a total of 6 gestures in 2 minutes, which I assume is far less than the number of actions (i.e. body movements, facial expressions, vocalizations) an infant would perform in 2 minutes. This poses several issues. First, the infant did not received a contingent response any time she performed an action, with a level of contingency experienced lower than in the other conditions.

REPLY: The reviewer’s comment - and description - concerning the design of our CR condition appears to indicate a misunderstanding of how this condition was deployed. With respect to the number of gestures performed by the experimenter in the 2 minutes of this experimental condition, we would like to point out that the experimenter performed a much higher number of gestures, with the total number of occurrences depending on how often each infant exhibited an action. As we explain in lines 239-241, the 3 actions were performed so that they matched the temporal features of the infants’ actions, and this included the temporal dynamics of an action (tempo, rhythmicity) when that action was repeated several times. This occurred often, and appears to be a common feature of infant behaviour at this age. Moreover, we were well aware that infants show a sensitivity to temporal features (and rhythms) from early in life. As such, if an infant performed an action (e.g. hit the table) four times, the experimenter responded with one of the actions from the inventory four times in a row. Thus the reviewer’s first concern that the infants did not receive a CR whenever performing an action does not apply.

Reviewer #2, concern (2): Second, by using one of the 3 predetermined responses, the infant received a random response to her actions, which could be contingent not only temporally, but also structurally and/or attuned in terms of affective valence, therefore resembling an imitative interaction more than a non-imitative contingent one. For example, if the infant waved her hand and by chance the imitator used as a response the ‘waving while saying Hello’ response, then the infant would experiences a temporal and structural contingency.

REPLY: To address the reviewer’s second concern, that the experimenter’s response could have matched the infant’s response both temporally and structurally, we specified in the revised manuscript (lines 248-256) that:

a) We considered this as a theoretical possibility, and thus the experimenter had the instruction to switch to a different action from the inventory if it was the case that the infant’s action by chance matched the experimenter’s action.
b) The chance that this would have occurred was from the start very low, as the behavioural repertoire of 6 month old infants does not typically include the actions performed by the experimenter. (And for what is worth, none of the infants in the study showed the behaviours). Six-month old infants typically do not wave and say ‘hej’, and mouth opening with a popping sound, although found to be frequently shown by mothers (e.g. Pawlby 1977), is not readily exhibited by infants. Indeed, a study found that 6-month infants require intensive training to learn a repeated mouth opening response (Kaye and Markus 1978).

Reviewer #2, concern (3 and 4): In addition, the presence of the vocalization would increase the salience of the response as well as its emotional content, thus resulting in a response very different from a non-imitative contingent response. Finally, the addition of a vocalization in all the responses provided by the imitator poses another methodological issue. In fact, whenever the infant performed an action in one modality only (e.g. a vocalization, a mouth gesture, a hand gesture), she would always receive a response in two different modalities, increasing the salience of the response and making the interaction more resembling a natural social interaction (as shown also by the results of the comparison between CR and the naturalistic mother-infant interactions).

If the scope of having a control condition is to show that temporal contingency alone is not effective in allowing infants to recognize they are being imitated and increasing their prosocial behaviours, then the variables mentioned above should be controlled more rigorously.

REPLY: In response to the reviewer’s third and fourth concern, we do not agree that responding with a bimodal action to potentially unimodal actions of the infant constitute an issue. This was a deliberate methodological choice grounded on empirical evidence that vocalising constitutes the most frequent form of maternal CR. We deliberately attempted to emulate this since mother-infant interaction is a context that confers ecological validity to CR conditions in general (for more details, see the reply to the comment below). Opting for a bimodal response was further grounded on evidence that bimodal stimuli have a perceptual advantage compared to unimodal stimuli. Finally, it was important that the CR condition was bimodal, considering that the MI and CI condition were occasionally bimodal. Bimodality was thus preferred in this design in order to ensure that the CR was sufficiently salient to such young infants, and thus the infants really had an opportunity to extract the temporal correspondence between the experimenter’s and their own actions. As a reminder, the essential feature of the CR condition is that infants control the “when” of the experimenter’s response. High signal salience does not change that during the CR condition the infant controls only the “when” of the experimenter’s responsiveness. Potential effects of bimodality on the perception of CR is, however, interesting as a topic of further research.

Reviewer #2 concern (5): Furthermore, to assess the efficacy of the CR condition, authors compared the levels of infant attention and smiling measured in this condition to those measured during a naturalistic mother-infant interaction. The two conditions are not comparable on any level, and it is not clear why the authors decided to make this comparison. If the scope was to see whether the interaction between the experimenter and the infant was affected by the non-familiarity with the imitator, then a comparison should be done between the interaction between mothers and infant and the preparation phase described on page 9. Alternatively, the authors could have asked the mothers to interact with their babies according to the same procedure used in the CR condition, thus making the two observations comparable.

REPLY: We believe this concern to be unfounded, considering that, as already mentioned above, mother-infant interaction is a context that provides ecological validity to standardised CR conditions, as deployed in imitation recognition studies. Moreover, as already mentioned in the original manuscript, in previous imitation recognition studies with infants, CR has received two different operationalisations: (i) regular mother-infant interaction; (ii) an experimenter responded with a temporally contingent but non-imitative action whenever the infant performed certain actions. The former operationalisation is present in studies with infants younger than the age targeted in our study, the second is present in studies with older infants. Thus this was one of the reasons why we compared the efficacy of our CR condition to regular mother-infant in an age-matched group. To further address the concern expressed by the reviewer, that mother-infant interaction (the naturalistic context of CR) and the CR condition (a standardised, experimental expression of CR) are “not comparable on any level”, we have expanded a number of paragraphs to emphasise that more than three decades of research have highlighted that mother-infant interaction is essentially characterised by CR, and that CR is a key feature of mother-infant interaction that promotes infant social-cognition. In other words, the whole point in adding CR as a control condition in imitation recognition studies is because infants are habitually exposed to CR in mother-infant interaction and the question is how they respond to a specific form of CR, i.e. sustained imitation. We have further discussed that a number of studies and theoretical papers stress that it is not merely the *temporal* aspect of maternal CR that is crucial to infant development, but the *form* as well. Our comparison thus shows that our CR condition (which is quite similar to previous designs used in imitation recognition studies with older infants, except for providing a higher level of CR since we did not make a selection concerning which actions should be responded to) was at least as efficient as mother-infant interaction (which is the operationalisation of CR in studies with younger infants) in eliciting social responsiveness from the infants. Sections relevant to the points summarised above are, at a minimum: lines 118-154; 257-277; 401-404

Reviewer #2 (concern 6): CI condition – this condition slightly differs from the MI one, as only the hand and trunk movements are imitated contralaterally, while all the facial gestures and vocalizations are imitated exactly as in the MI condition. The similarity between the two conditions might explain why no difference has been found in any of the infant behaviours between the MI and the CI conditions.

REPLY: This description is correct, and is also consistent with the purpose of the condition, i.e. to control for the effect of spatial compatibility, all other things remaining equal. In contrast, in the BI condition, we assess the effects of emotional (facial, vocal) feedback during imitation, by removing such responses. Previous studies on imitation recognition in infants have not controlled for spatial compatibility effects, but studies with adults point to spatial compatibility as a potential moderator of imitation recognition and imitation-induced prosociality (as explained in lines 157-169). In this study, we take a first stab at testing spatial compatibility effects on imitation recognition in infants. Further research can introduce additional manipulations. During pilot testing we had several other manipulations in mind, but it soon became clear that extending the duration of the experimental phase beyond 10 minutes was problematic with such young infants, especially given the variation in behaviours across the four experimental intervals and the four SF intervals.

Reviewer #2 (concern 7): Still face interval – the duration of the still-face phase should be similar to the one used for the other experimental conditions (i.e. 2 minutes). Even if the length of the still-face phase has varied between 30 and 180 seconds in previous studies, for a more rigorous experimental design and in order to investigate the still-face effect on infant behaviours, the same duration should be used for the interactive phase and the still-face one.

REPLY: To address the reviewer’s concern we revised the manuscript to further stress that the SF-intervals are not experimental conditions, and that the purpose of this study is not to study the still-face (SF) effect. The purposes of this study was to assess imitation recognition in 6-month old infants and the effects of being imitated on infant social responsiveness and prosocial inclination. The SF-intervals are used as a tool for eliminating carryover effects from one experimental condition to another. As a consequence we tailored the duration of the SF intervals to suit the purposes of the study while precluding counter-productive SF effects. As detailed in lines 317-333, the duration of 30 seconds was based on both pilot testing and findings from previous studies concerning the carryover effects of the SF itself. We explained that we wanted to avoid unnecessarily long SF intervals as we wanted to avoid carryover effects from the SF intervals to the experimental conditions. Such effects are documented in the literature to vary with the duration of the SF, with longer durations requiring longer recovery times once the mother / experimenter becomes active again. We observed such effects in our pilot testing too. Moreover, we also sought to keep the overall duration of the testing to no longer than 10 minutes, as longer durations appeared to be too straining for such young infants, especially with repeated SF intervals.

Reviewer #2 (concern 8): Moreover, similarly to previous studies, the authors should include non-distress vocalizations among the behaviours scored during the still-face phase, as it have been shown that infant increase the frequency of such behaviours after an imitative interaction in the attempt to re-engage the interaction with the imitator.

REPLY: We agree with the reviewer that analysing the infants’ responses during the SF intervals could be potentially revealing, and we now include this point and a discussion in the revised manuscript (lines lines 333-346; 387-397; 790-801; 1012-1019). Indeed, it has been speculated that these could provide an additional measure on the effects of condition. This speculation is based on findings suggesting that infants’ social responsiveness during SF intervals is susceptible to effects of previous engagements, at least in the long term. We are aware of reports that non-distress vocalisations during the SF appear to be higher in the infants of mothers who exhibit a higher level of maternal mirroring (Bigelow and Walden 2009; Bigelow et al. 2018), but to our knowledge this has not been measured in any imitation recognition study that contrasted imitation with e.g. CR. Three remarks are in place here: (i) maternal mirroring is not to be confused with imitation, as maternal mirroring is a composite construct that overlaps much with the notion of affect attunement, and refers to maternal behaviours that create shared affect, e.g. facial mimicry, vocal mimicry, cross-channel emotional mimicry; (ii) the effects previously reported for higher levels of maternal mirroring could be longer term effects, i.e. a consequence of interactional history, and of the fact that infants have been consistently exposed, over a long time, to higher maternal mirroring; (iii) non-distress vocalisations are a form of social bidding; in other studies, social bidding have been more broadly operationalised to include even other behaviours directed to the mother / experimenter by which the infant attempted to re-engage the adult in interaction. In this respect, one previous study that contrasted imitation and CR measured this type of social bidding and found no differences in the amount of social bidding directed to the imitating vs the CR experimenter (Agnetta and Rochat, 2004). An additional study with macaque infants measured attention and affiliative signals (lip smacking) in the SF following an imitation condition and non-CR condition (i.e. the experimenter’s actions were not contingent upon those of the macaque infant) and found that both attention and lip smacking were higher after being imitated (Sclafani et al. 2015). Since the control condition was not CR it is not possible to compare this study with our study, nor with Agnetta and Rochat 2004. Moreover, Agnetta and Rochat failed to find differences in the levels of attention and smiling that the infants direct to the imitating and CR experimenter.

Reviewer #2 (concern 9): The authors should have coded infants’ social and testing behaviours in both the interactive phase of the interaction and the still-face one, keeping the two phases separated. From the analysis it is not clear whether the behaviours scored in the 30 sec of still face were not included in the analysis or were scored together with the first 2 minutes of interaction.

An analysis of the behaviours shown by the infants during the still-face period would allow authors to better understand whether infants are sensitive to the quality of the interaction therefore showing more prosocial behaviours toward the imitator as well as whether they perceive themselves as active agents within the interactions by showing more social bids and testing behaviours.

REPLY: The results presented in the original manuscript reflected infant responsiveness during the experimental intervals, and this is specified now in the Results sections, and in the relevant headings. We did not include an analysis of responses in the SF intervals, since, as already stated, the purpose of this study was not (and is not) to study the SF-effect, i.e. the SF-intervals are not to be equated with experimental conditions. As discussed above, responsiveness during the SF intervals has been contemplated as a potential additional measure of imitation recognition. However, based on a previous study (Agnetta and Rochat 2004), this appears not to be a useful measure, as in that study there was no difference in the levels of social responses directed towards the imitating vs. the CR experimenter during the SF. We have conducted similar analyses, and these are now included in the manuscript (lines 718-847), and pages 19-35 in the Supplementary results. Our results are consistent with Agnetta and Rochat (2004) in that the condition had no effect on the level of social responsiveness in the SF intervals. We have found, on the other hand, order effects, with certain responses being higher in the first SF interval compared with each subsequent SF interval. We have also analysed social bidding, but given the low number of non-distress vocalisations, we opted for a cumulative measure similar to Agnetta and Rochat (2004). Once again, there was no effect of condition, but there was an order effect, whereby social bidding was higher during the first SF interval compared with the last SF interval. Higher social responsiveness in the first SF interval is consistent with results from previous studies that included repeated SF-intervals. These additional results are now also discussed in the Discussion section (lines 1002-1022).

3. RESULTS AND DISCUSSION

Reviewer #2 (concern 10): Overall, the authors should discuss each of the results found more extensively and with more support.

In particular, for all the infant behaviours included in the analysis they should further discuss the similarity between the MI condition and the CI one. As stated before, the two conditions differ very slightly to each other, and this could explain why no difference was found. Alternatively, results should be discussed in term of structural resemblance in both conditions despite the inversion in the left-right axis.

REPLY: We would like to point out that this concern was already addressed in our original manuscript. In lines 868-884 of the Discussion we cover the need to control for spatial compatibility effects in imitation recognition studies with infants (which is also discussed in lines 157-169 of the Introduction section). In the following paragraph of the Discussion section (lines 885-892), we comment that the configural manipulation (introduced in the CI condition) did not affect implicit and explicit imitation recognition. We also discuss that, in spite of the left-right axis inversion, the infants appear to have detected the structural similarity between their own and the experimenter’s actions. We made a small edit in the revised manuscript in order to remind the reader that the ‘configural manipulation’ refers to left-right axis inversion.

Reviewer #2 (concern 11): I agree with the decision of scoring infant behaviours in term of duration, frequency, and mean duration of each bout, as different measures can indeed provide different information about the interaction. However, authors did not discuss their findings in this regard. For example, they found that in MI and CI conditions infants looked more to the experimenter, less frequently but longer in each bout, and discussed this in terms of implicit recognition and more social engagement during the imitative interactions with high levels of contingency. However, they did not discuss that infants in BI and CR, despite showing less social engagement, looked more frequently at the experimenter, suggesting evidence for monitoring behaviours towards him/her.

Similarly, infants smiled longer in the MI and CI conditions, but the frequency of smiles was not different among the four different conditions, suggesting again an effect of imitation on social engagement but also a possible use of social signals in trying to engage socially with the experimenter in the other two conditions. In this regard, analyzing the data according to the experimental phase (interaction vs still face) would give more information about the quality of the interaction and the active role of the infant in modulating the interaction.

REPLY: We have addressed this concern by adding a paragraph (lines 893-913 that covers the similarities between the MI and CI condition with respect to patterns of social responsiveness. We interpreted the higher frequency of attention bouts during BI and CI as indicating a more fragmentary social engagement. As commented by the reviewer, we add that this suggests that the infants continued to monitor (and briefly interact with) the experimenter. However, the shorter duration of attention bouts can be an indication that the infants likely found these types of interactions less interesting / engaging. In the same paragraph, we further discuss the differences related to smiling patterns. In particular, the longer duration of smiling during MI and CI and the absence of a condition effect on smiling frequency suggests that infants’ affective engagements were tentative and fleeting in the BI and CR conditions compared to the MI and CI conditions.

Reviewer #2 (concern 12): Another interesting result that would be worth discussing further is that infants showed the same amount and duration of testing behaviours in the MI, CI and BI conditions. Interestingly, the suppression of emotional mimicry lead to a decrease in social engagement, but did not prevent infant to detect behavioural similarities between their own actions and those performed by the experimenter and show testing behaviours toward him.

REPLY: We would like to point out that this aspect is already discussed in the original manuscript. Since the reviewer made this comment, however, we judged that it would be useful to further clarify this aspect, and thus edited the paragraph accordingly (lines 9145-933).

Reviewer #2 (concern 13): Again, it would be interesting to see whether the same results would be obtained when scoring testing behaviours during the two phases of the interaction.

REPLY: This comment is a bit unclear. We interpret it as suggesting a comparison between the experimental condition and the SF intervals. Following the suggestion of the reviewer, for the SF intervals we analysed levels of attention, smiling, approach and social bidding during the SF intervals (with results being reported in lines 718-847, and pages 19-35 of the *Supplementary results*). As we point out in lines 388-390), we did not score testing behaviours during the SF intervals since the experimenter is inactive, and thus, there are no behaviours to be subjected to testing by the infants.

In the revised version of the manuscript we have added a paragraph in the Discussion section (lines 1002-1022) in which we discuss patterns of responsiveness during the SF intervals (with respect to attention, smiling, approach and social bidding), as well as patterns of responsiveness comparatively between the experimental conditions and the SF intervals (with respect to attention, smiling, and approach - as these are the tree responses that can be compared). We note that patterns of responsiveness go in opposite directions in the experimental conditions compared to the SF intervals. Patterns of responsiveness during the experimental conditions exhibit condition effects, but no order effects. Patterns of responsiveness during the SF intervals exhibit order effects, but no condition effects. In particular, infants showed higher levels of social responses in the first SF interval compared to each subsequent SF interval. We comment that these results suggest that the infants quickly extracted the temporal organisation of the experimental phase, which includes an alternation of activity and inactivity phases, which explains why levels of social engagement were higher during the first SF interval, when this was novel. Approach behaviours occurred seldomly during the SF intervals, and thus the results are inconclusive. With so few occurrences, statistical treatment is likely to be meaningless. (We have, nevertheless conducted tests even on such small datasets, and these are reported in the Supplementary results file) For social bidding we only found a difference between the first SF interval compared to the last SF interval. These results could too be affected by the small number of occurrences, with the possibility to detect further differences with a larger sample.

Minor issues:

Reviewer #2 (concern 14): The introduction would benefit from the inclusion of more previous literature on imitation recognition and maternal mirroring in young infants and non-human primates (e.g. Beebe et al., 2003; Meltzoff, 2007; Murray et al., 2014; Murray et al., 2016; Sclafani et al., 2014).

REPLAY: We have included Murray et al. 2016 and Sclafani et al. 2015, as the studies report a number of findings that are relevant for the purposes of our study. We did not include Meltzoff 2007, as the relevant theoretical points made in this paper are also present in two earlier papers that are already cited in the manuscript. With respect to Beebe et al. 2003, we are unsure which of Beebe et al. 2003 studies is suggested by the reviewer. There are several Beebe et al. 2003 studies potentially relevant to the manuscript, yet we believe that none of these provide additional points for the discussion. We are similarly unsure about which Murray et al. 2014 study is referred to above.

Reviewer #2 (concern 15): Table 4 – Check the frequency of attention in the CR experimental condition and the control mother-infant interaction. The value reported are 12.438 (1.021) and 1.355 (12.065) respectively. I assume the second value is not correct, as no difference was found between the two conditions, and considering the extremely high SEM value.

REPLY: We are grateful for this observation. This has now been corrected.

Reviewer #2 (concern 16): Authors should explain why they used the Benjamini-Hochberg correction for multiple comparisons instead of the Bonferroni one.

REPLY: To address this comment, we now clarify that we use Benjamini-Hochberg since this correction reaches a better compromise between limiting false positives and power reduction (lines 441-442). Bonferroni, which historically has had a wider use, sacrifices the last aspect, and thus a number of significant differences could be missed. As usual, significant differences should be read and interpreted in concert with reported effect sizes.
